# Supplementary figures and images for: SRSF6 balances mitochondrial-driven innate immune outcomes through alternative splicing of BAX
Source: eLife. 2022 Nov 21;11:e82244. doi: 10.7554/eLife.82244 (PMC9718523; doi:10.7554/eLife.82244)

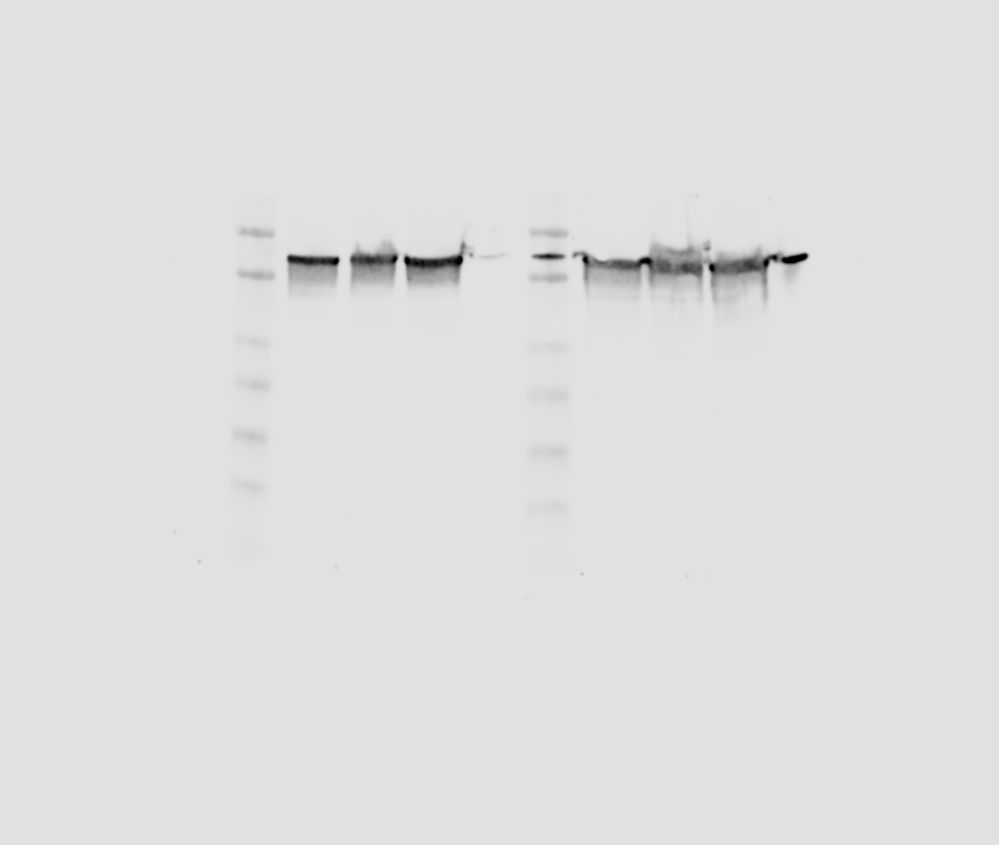

Supplement: Figure 1—source data 1. — Unmodified immunoblots of RSAD2 (VIPERIN) and TUBULIN in Srsf6 KD RAW MΦ. As in G but for phosphorylated IRF3, total IRF3, and ACTIN. Boxed bands indicate what is shown in the main figures. Arrows indicate bands of interest. [file elife-82244-fig1-data1.zip › Source Data Figure 1/Source Data Figure 1L IRF3.png]

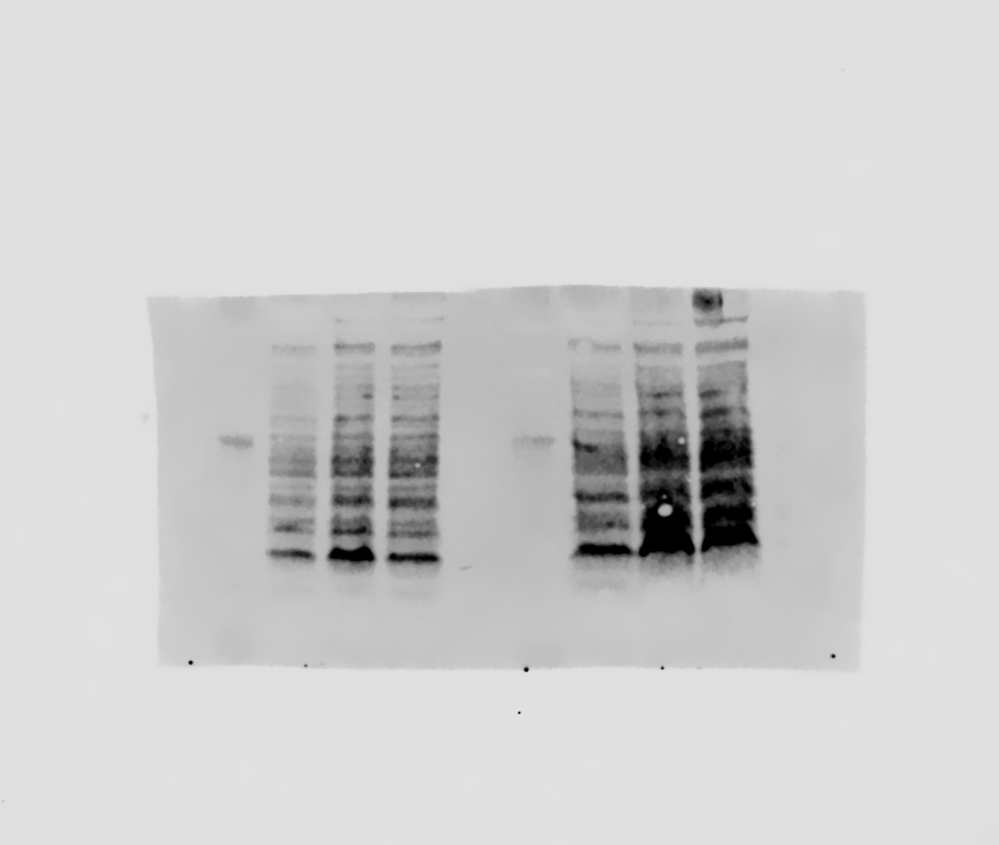

Supplement: Figure 1—source data 1. — Unmodified immunoblots of RSAD2 (VIPERIN) and TUBULIN in Srsf6 KD RAW MΦ. As in G but for phosphorylated IRF3, total IRF3, and ACTIN. Boxed bands indicate what is shown in the main figures. Arrows indicate bands of interest. [file elife-82244-fig1-data1.zip › Source Data Figure 1/Source Data Figure 1L pIRF3.png]

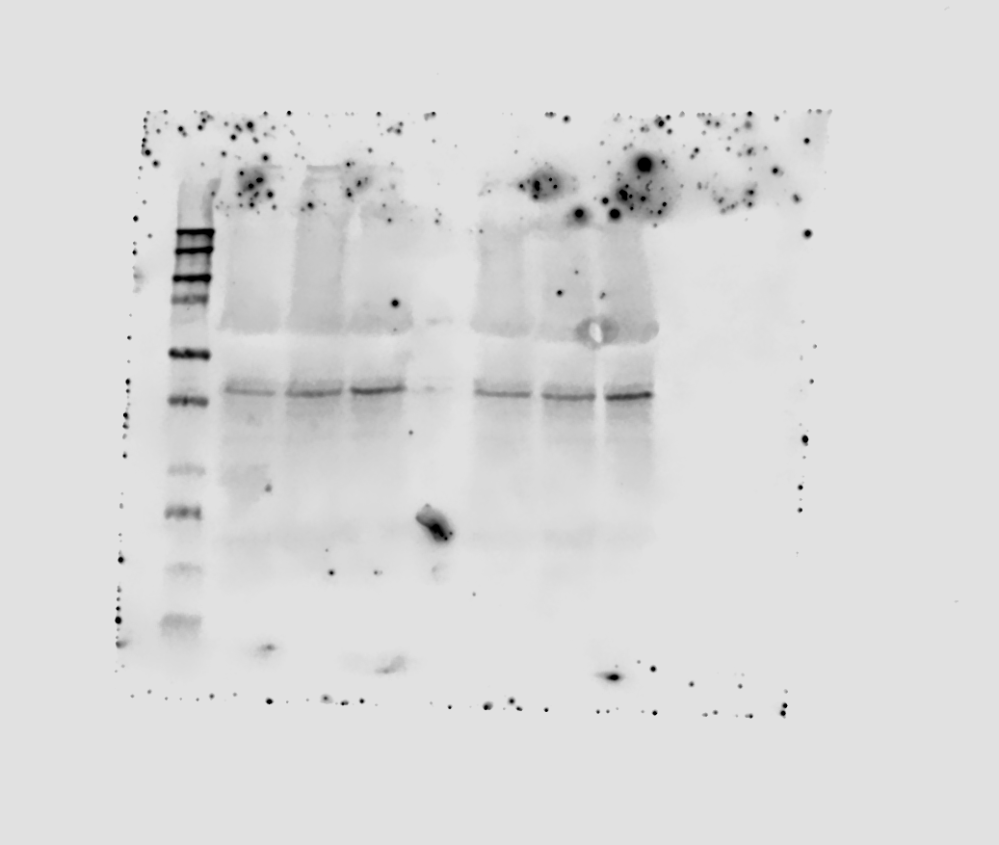

Supplement: Figure 1—source data 1. — Unmodified immunoblots of RSAD2 (VIPERIN) and TUBULIN in Srsf6 KD RAW MΦ. As in G but for phosphorylated IRF3, total IRF3, and ACTIN. Boxed bands indicate what is shown in the main figures. Arrows indicate bands of interest. [file elife-82244-fig1-data1.zip › Source Data Figure 1/Source Data Figure 1J Rsad2.png]

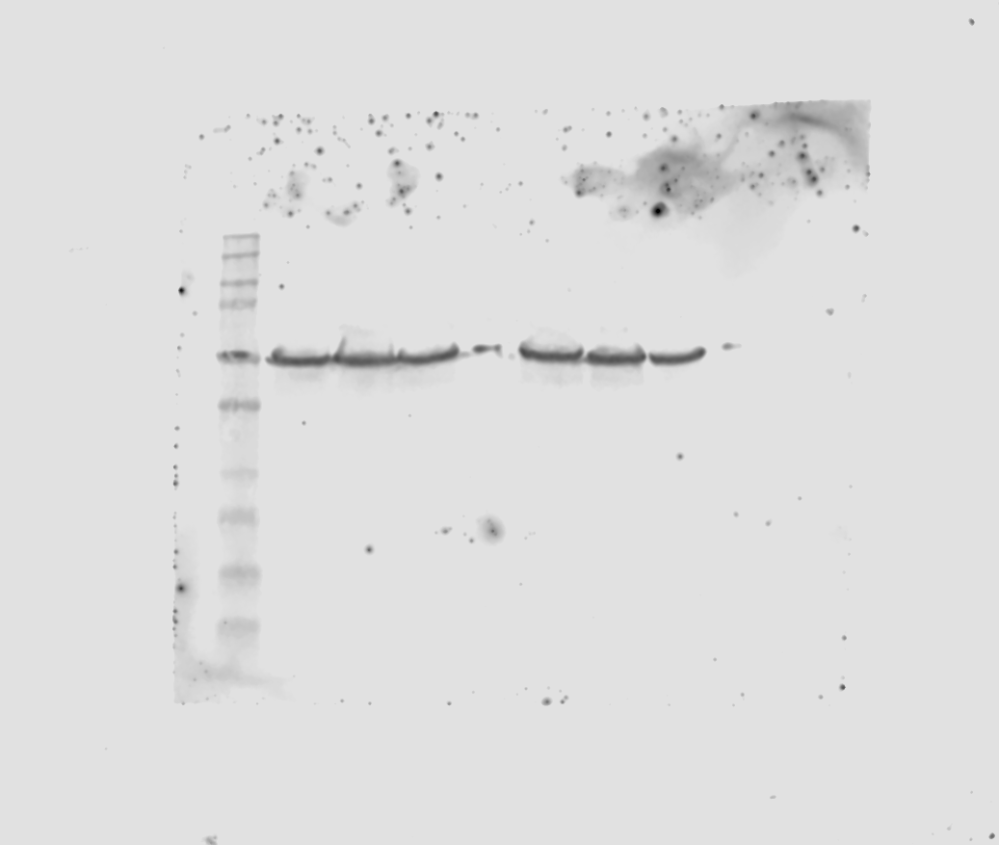

Supplement: Figure 1—source data 1. — Unmodified immunoblots of RSAD2 (VIPERIN) and TUBULIN in Srsf6 KD RAW MΦ. As in G but for phosphorylated IRF3, total IRF3, and ACTIN. Boxed bands indicate what is shown in the main figures. Arrows indicate bands of interest. [file elife-82244-fig1-data1.zip › Source Data Figure 1/Source Data Figure 1J Tubulin.png]

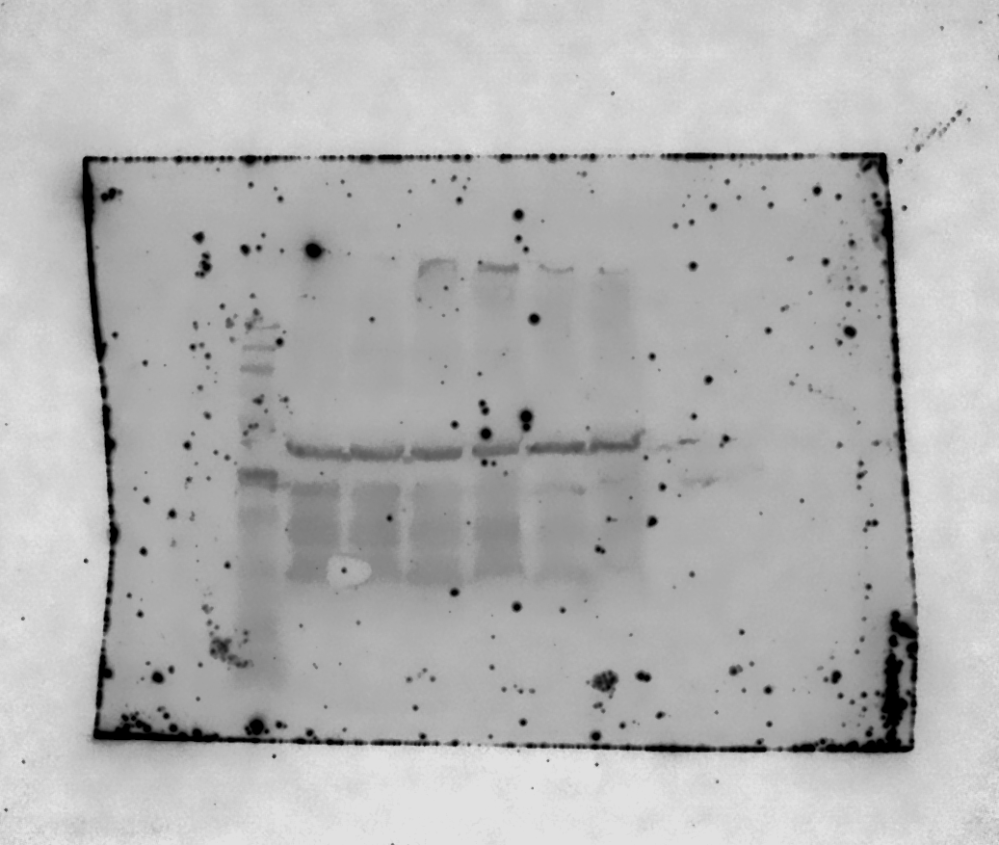

Supplement: Figure 1—source data 1. — Unmodified immunoblots of RSAD2 (VIPERIN) and TUBULIN in Srsf6 KD RAW MΦ. As in G but for phosphorylated IRF3, total IRF3, and ACTIN. Boxed bands indicate what is shown in the main figures. Arrows indicate bands of interest. [file elife-82244-fig1-data1.zip › Source Data Figure 1/Source Data Figure 1G Actin.png]

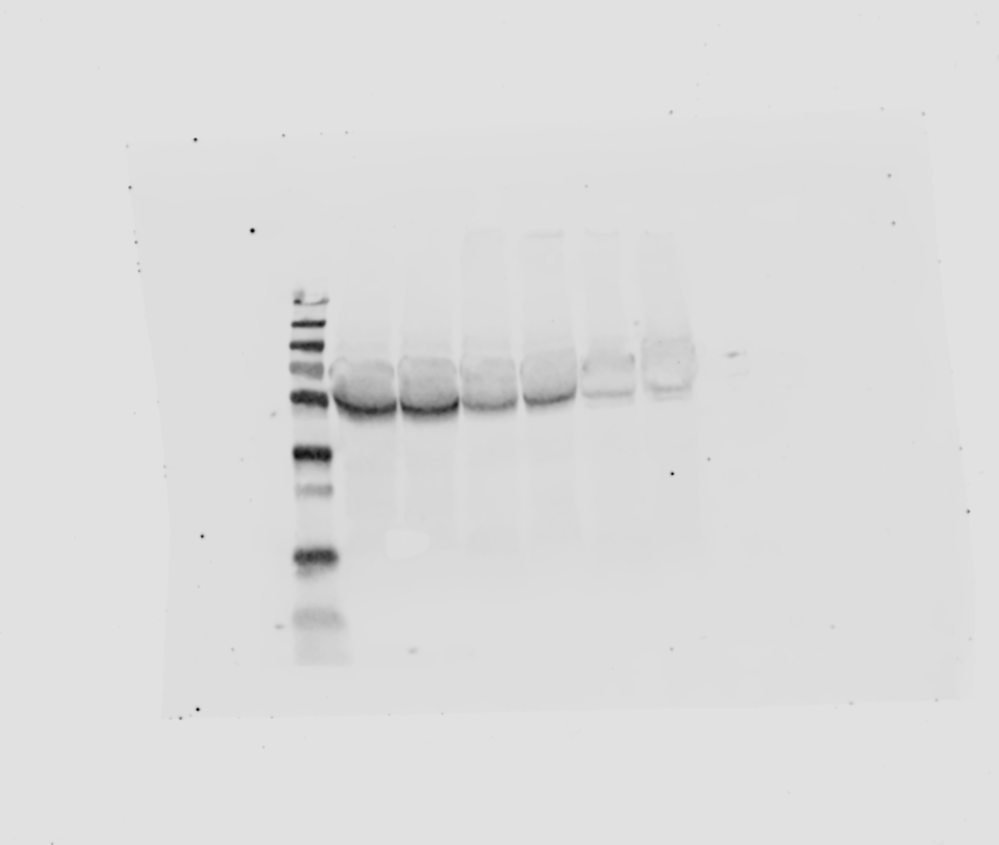

Supplement: Figure 1—source data 1. — Unmodified immunoblots of RSAD2 (VIPERIN) and TUBULIN in Srsf6 KD RAW MΦ. As in G but for phosphorylated IRF3, total IRF3, and ACTIN. Boxed bands indicate what is shown in the main figures. Arrows indicate bands of interest. [file elife-82244-fig1-data1.zip › Source Data Figure 1/Source Data Figure 1G Srsf6.png]

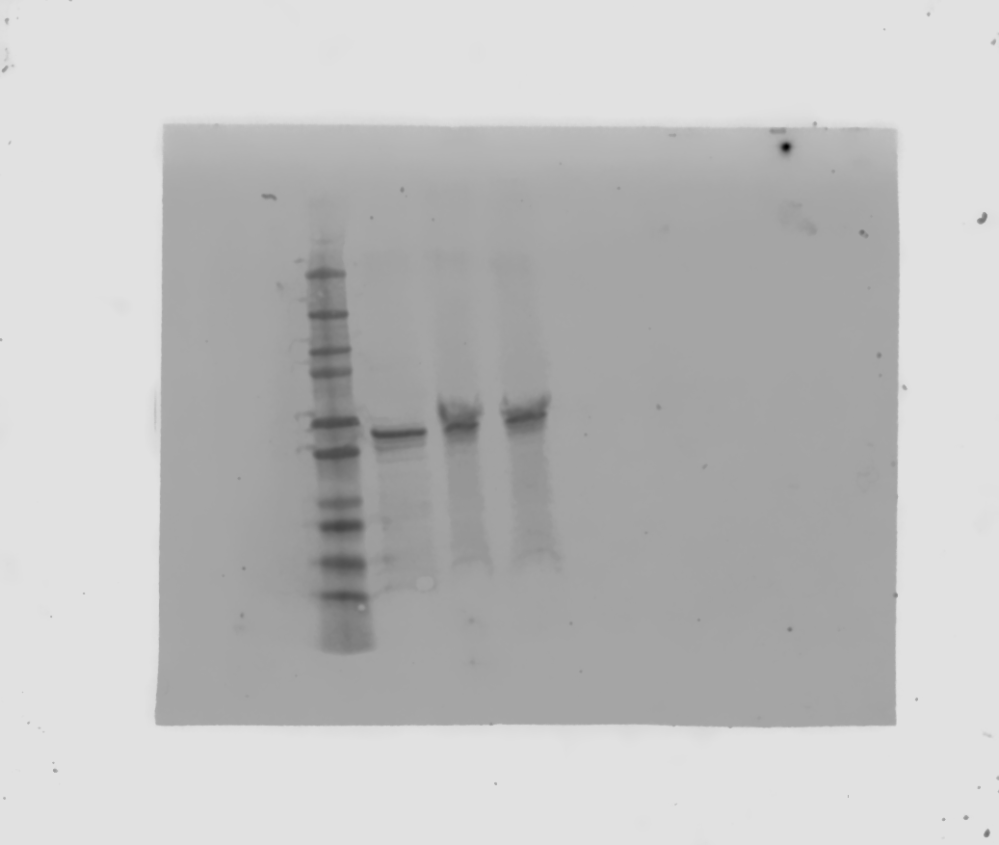

Supplement: Figure 1—source data 1. — Unmodified immunoblots of RSAD2 (VIPERIN) and TUBULIN in Srsf6 KD RAW MΦ. As in G but for phosphorylated IRF3, total IRF3, and ACTIN. Boxed bands indicate what is shown in the main figures. Arrows indicate bands of interest. [file elife-82244-fig1-data1.zip › Source Data Figure 1/Source Data Figure 1L Actin.png]

G

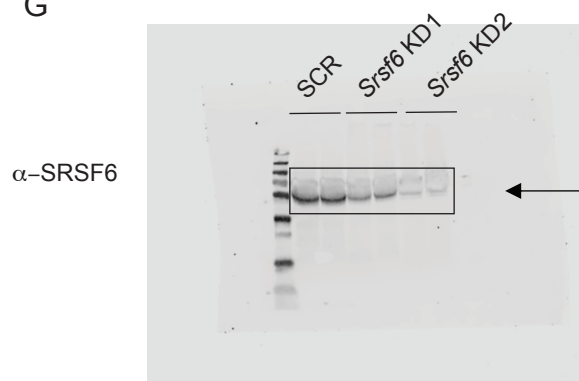 $\alpha$ -ACTIN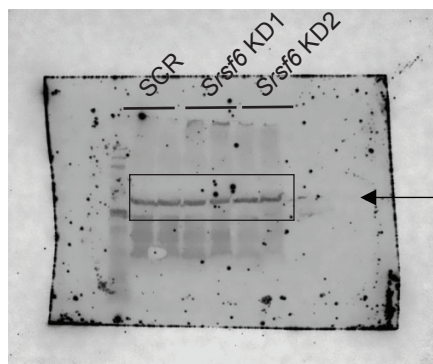

J

 $\alpha$ -RSAD2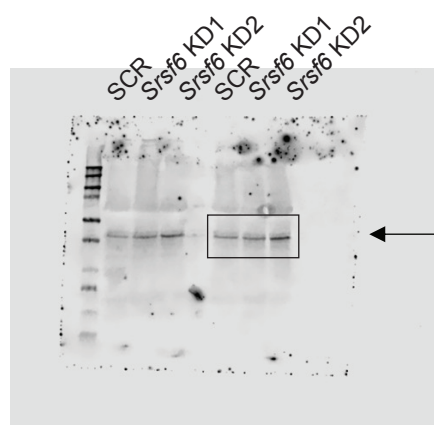 $\alpha$ -TUBULIN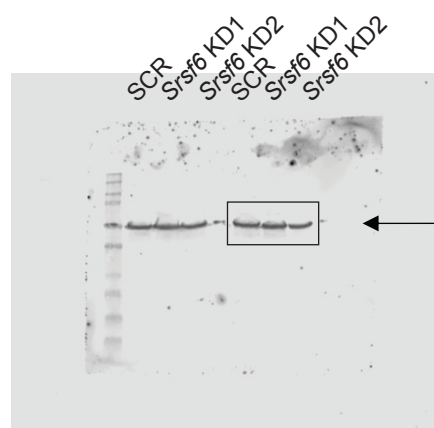

L

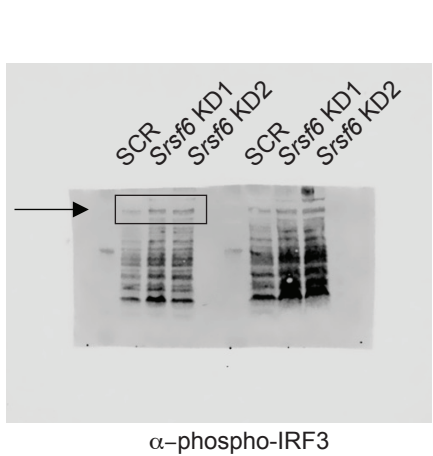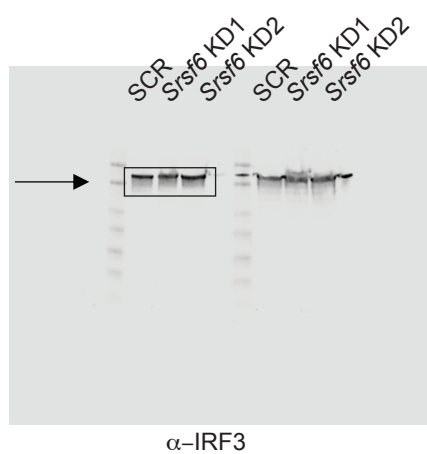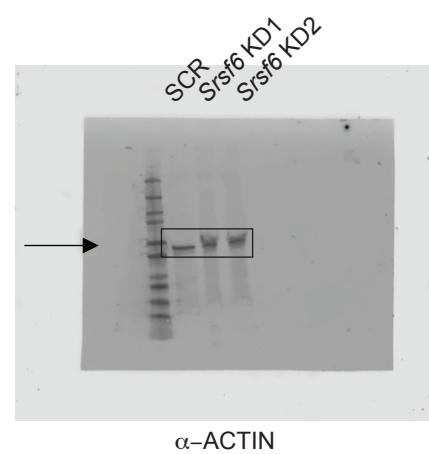

Figure 1. Source Data

Supplement: Figure 1—source data 1. — Unmodified immunoblots of RSAD2 (VIPERIN) and TUBULIN in Srsf6 KD RAW MΦ. As in G but for phosphorylated IRF3, total IRF3, and ACTIN. Boxed bands indicate what is shown in the main figures. Arrows indicate bands of interest. [file elife-82244-fig1-data1.zip › Source Data Figure 1/eLIFE Figure 1, Source Data 1.pdf]

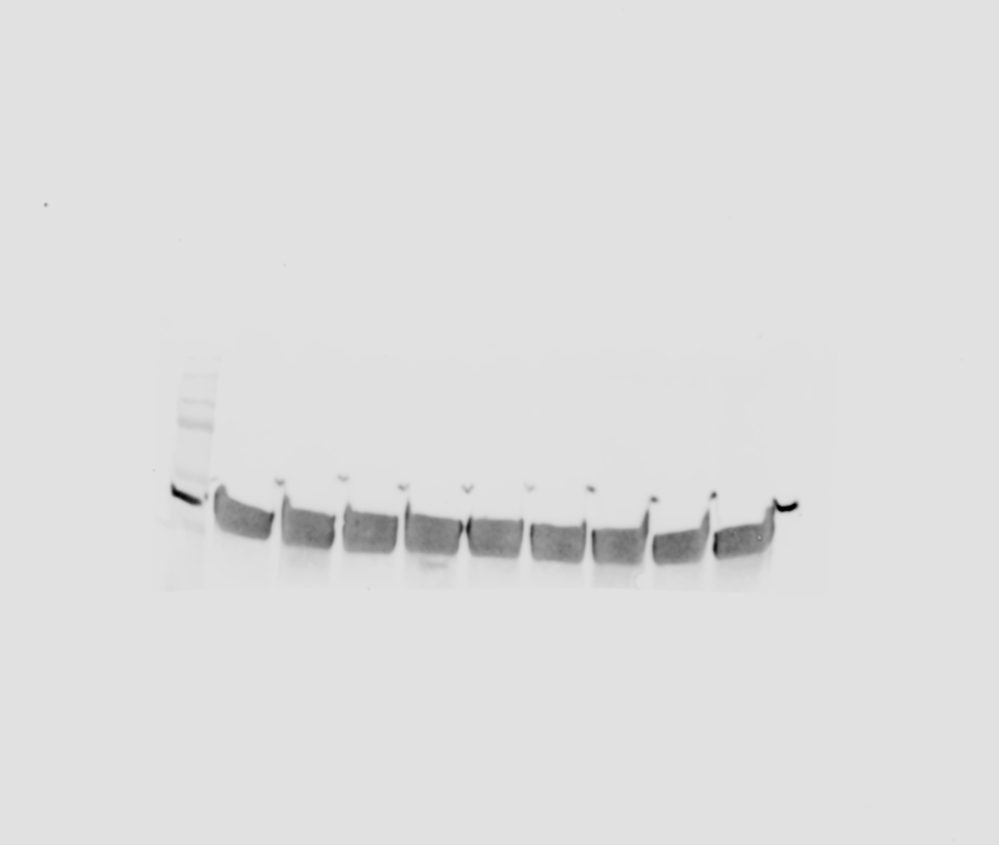

Supplement: Figure 2—source data 1. [file elife-82244-fig2-data1.zip › Source Data Figure 2/Source Data Figure 2A Total Actin.png]

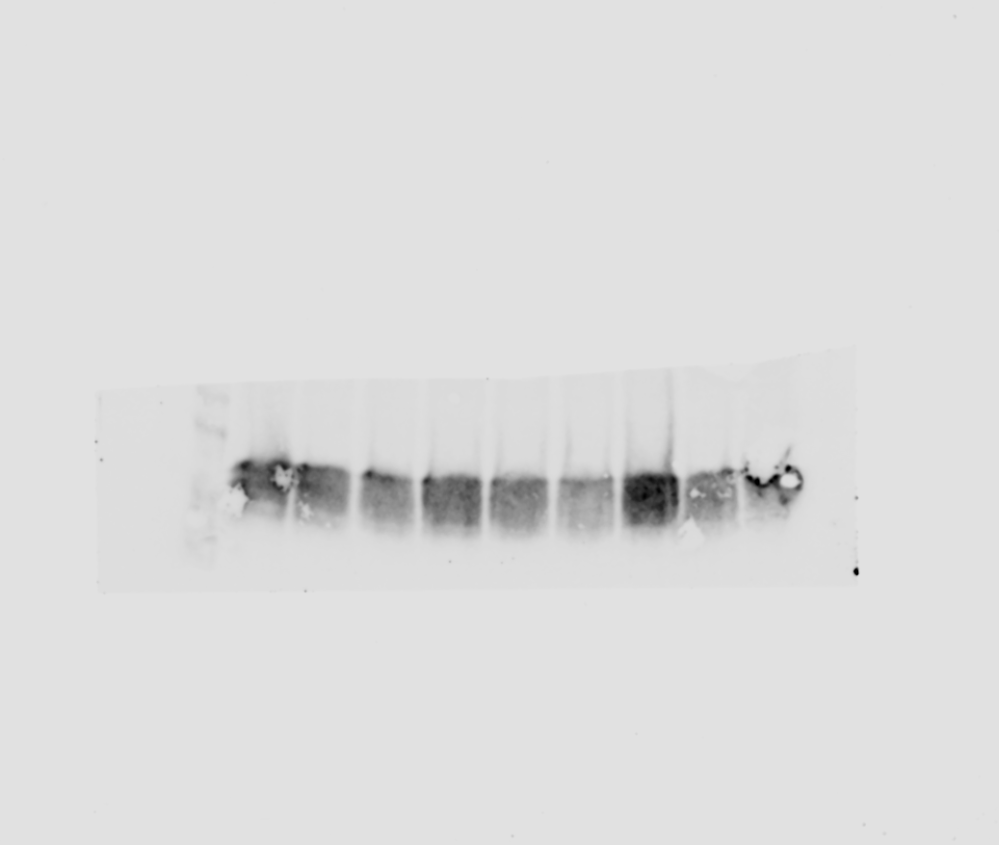

Supplement: Figure 2—source data 1. [file elife-82244-fig2-data1.zip › Source Data Figure 2/Source Data Figure 2A Membrane Tom20.png]

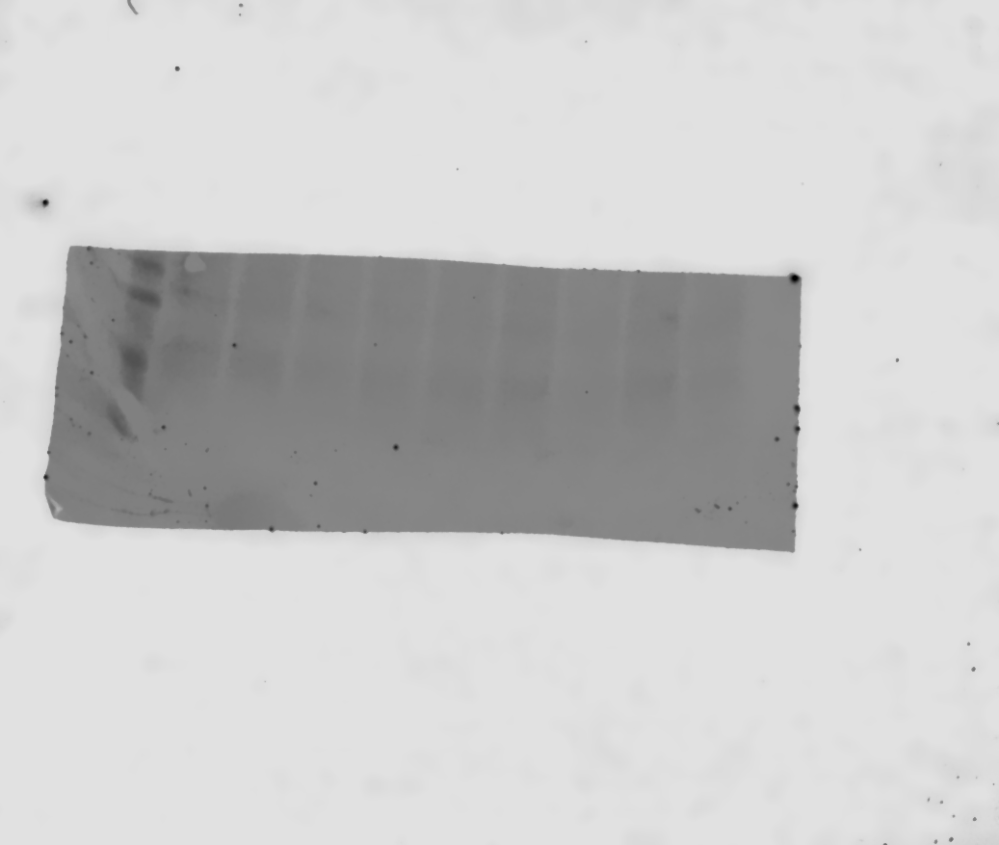

Supplement: Figure 2—source data 1. [file elife-82244-fig2-data1.zip › Source Data Figure 2/Source Data Figure 2A Cyto Tom20.png]

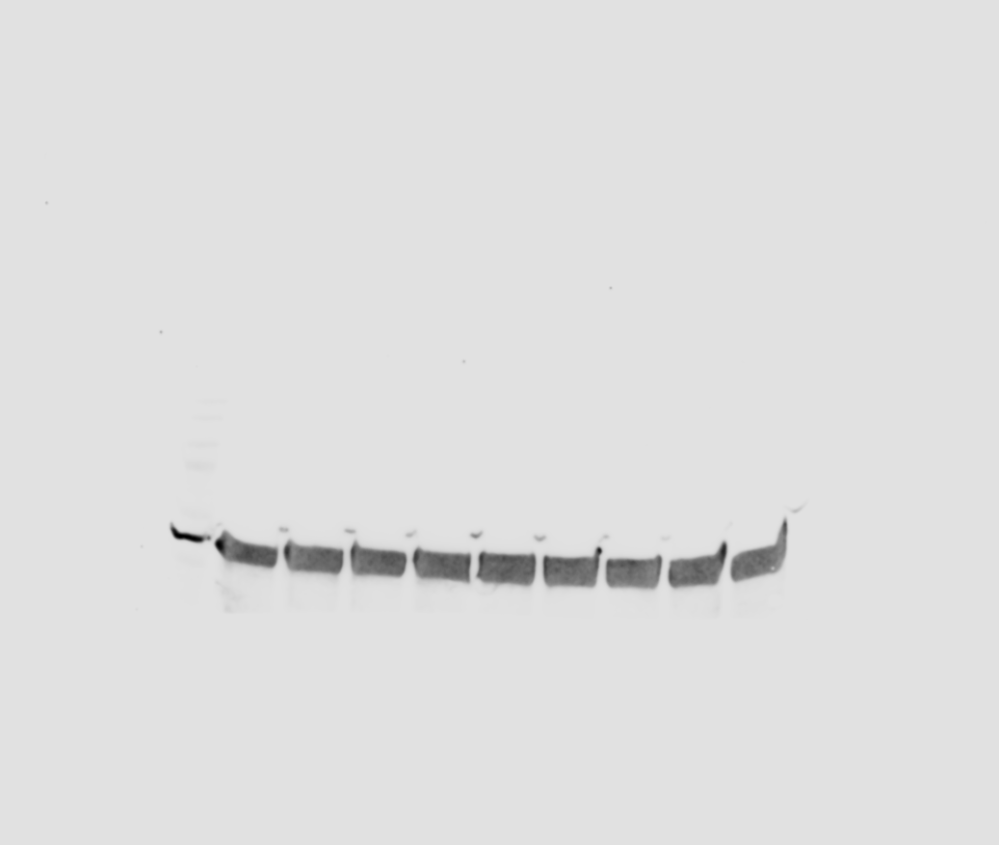

Supplement: Figure 2—source data 1. [file elife-82244-fig2-data1.zip › Source Data Figure 2/Source Data Figure 2A Cyto Actin.png]

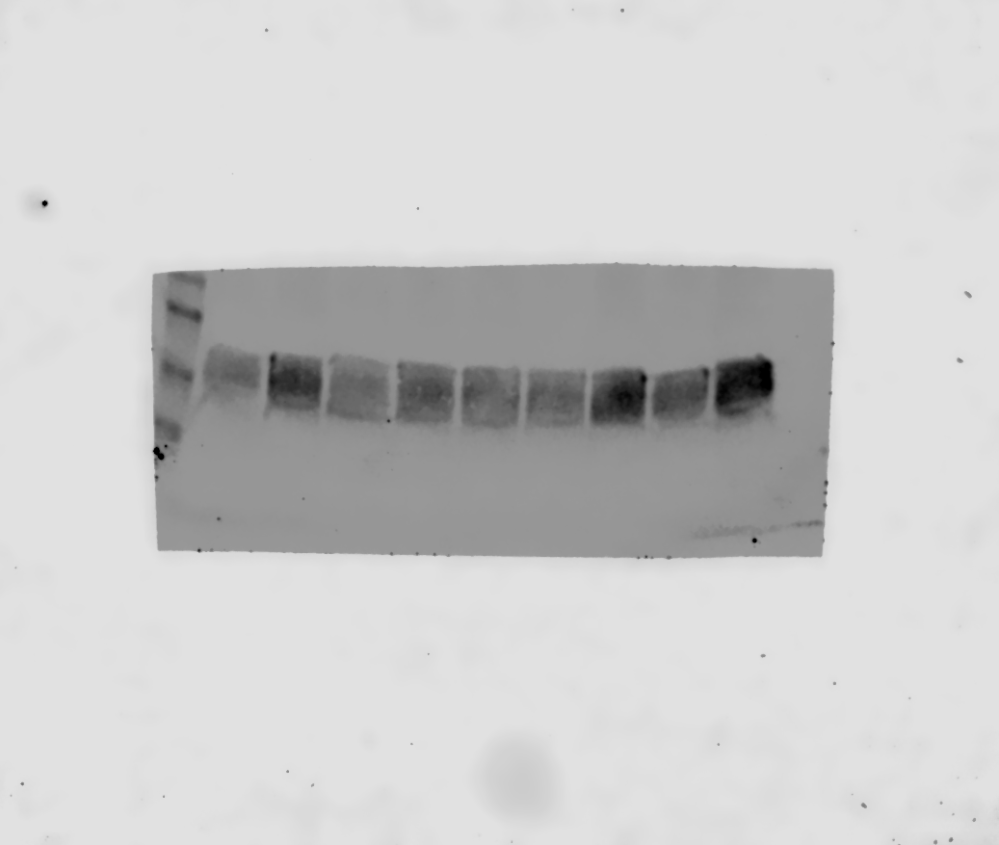

Supplement: Figure 2—source data 1. [file elife-82244-fig2-data1.zip › Source Data Figure 2/Source Data Figure 2A Total Tom20.png]

A

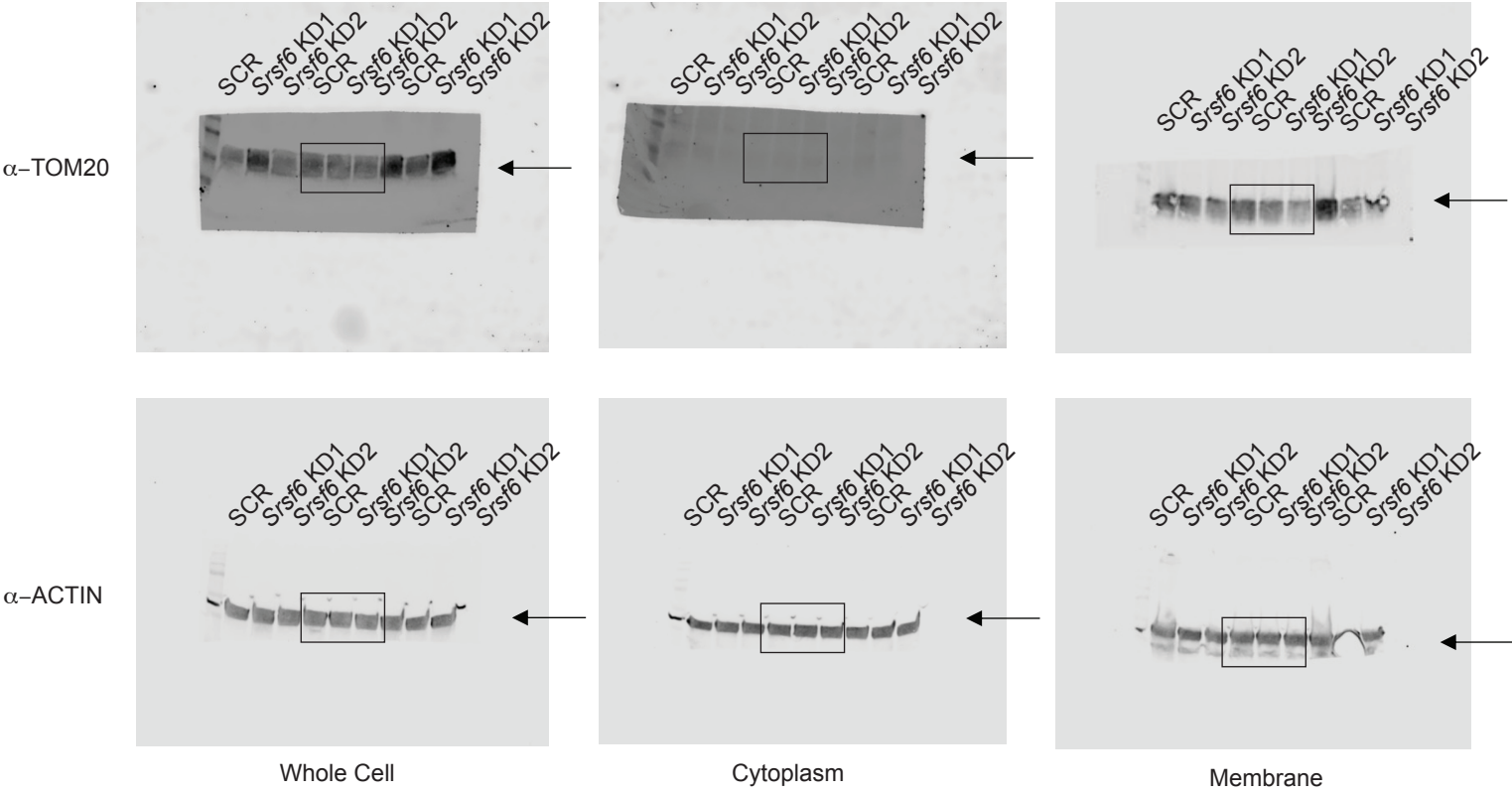

\* last three lanes treated with ABT737 and Q-vD-OPH

Figure 2. Source Data

Supplement: Figure 2—source data 1. [file elife-82244-fig2-data1.zip › Source Data Figure 2/eLIFE Figure 2, Source Data 1.pdf]

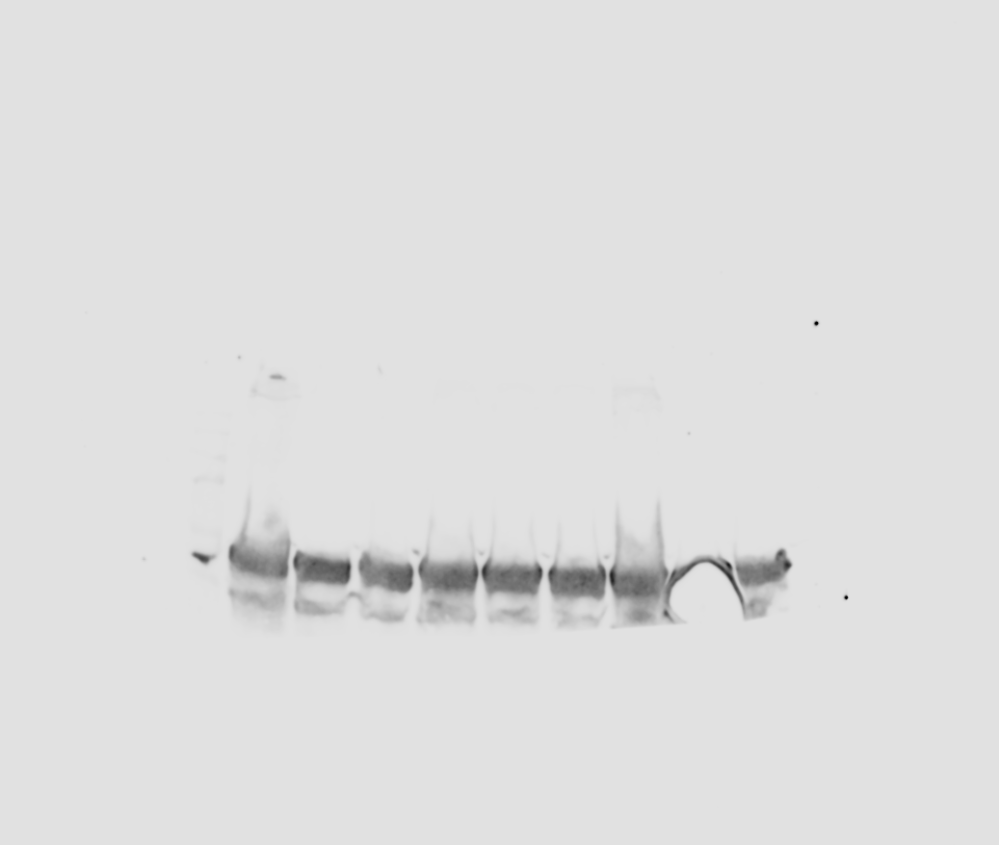

Supplement: Figure 2—source data 1. [file elife-82244-fig2-data1.zip › Source Data Figure 2/Source Data Figure 2A Membrane Actin.png]

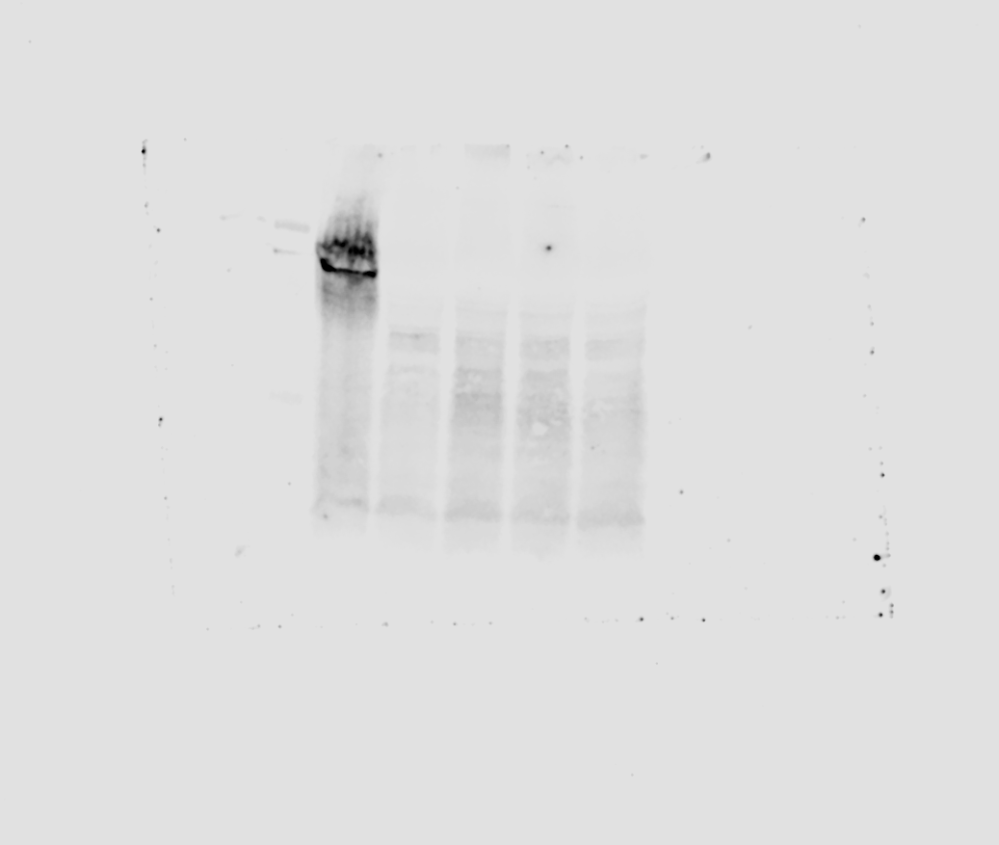

Supplement: Figure 2—figure supplement 1—source data 1. — cGAS lanes are from multiple protein preparations. Boxed bands indicate what is shown in the figure. Arrows indicate bands of interest. [file elife-82244-fig2-figsupp1-data1.zip › Figure supplemental 2A cGAS.png]

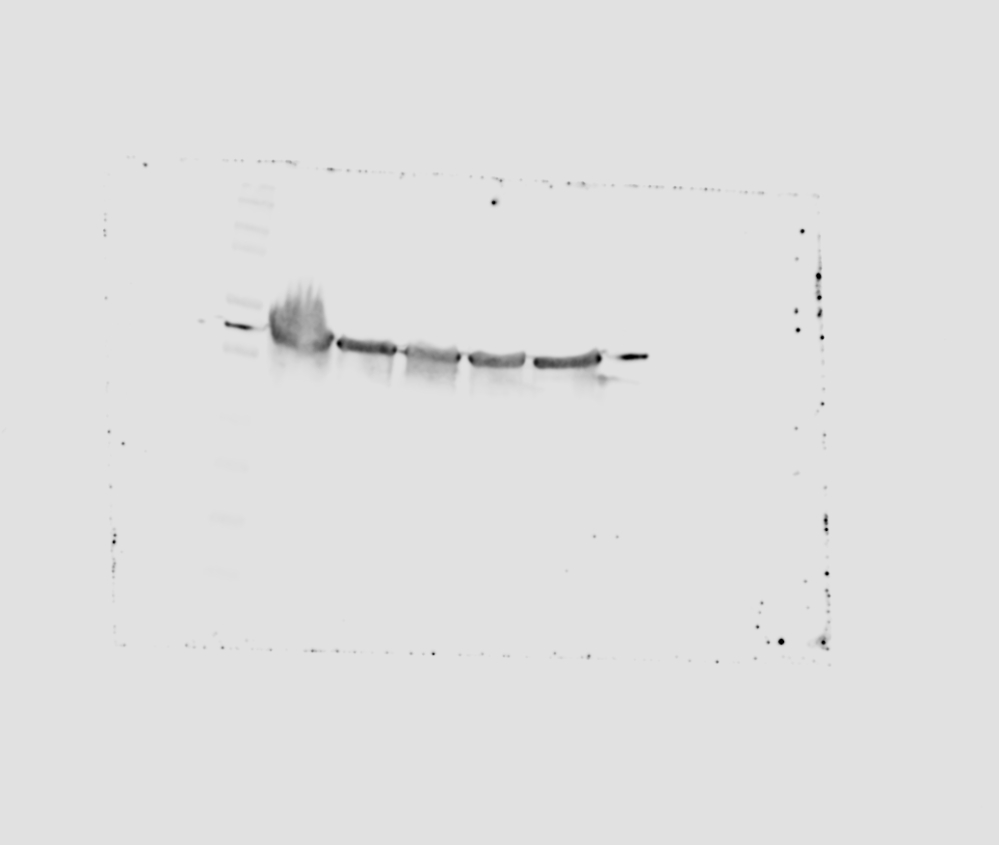

Supplement: Figure 2—figure supplement 1—source data 1. — cGAS lanes are from multiple protein preparations. Boxed bands indicate what is shown in the figure. Arrows indicate bands of interest. [file elife-82244-fig2-figsupp1-data1.zip › Figure suppplemental 2A Actin.png]

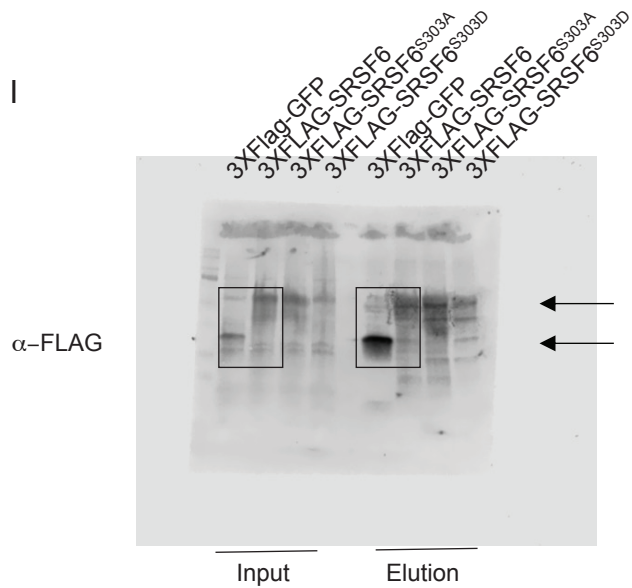

Figure 3. Source Data

Supplement: Figure 3—source data 1. [file elife-82244-fig3-data1.zip › Source Data Figure 3/eLIFE Figure3 Source Data_New.pdf]

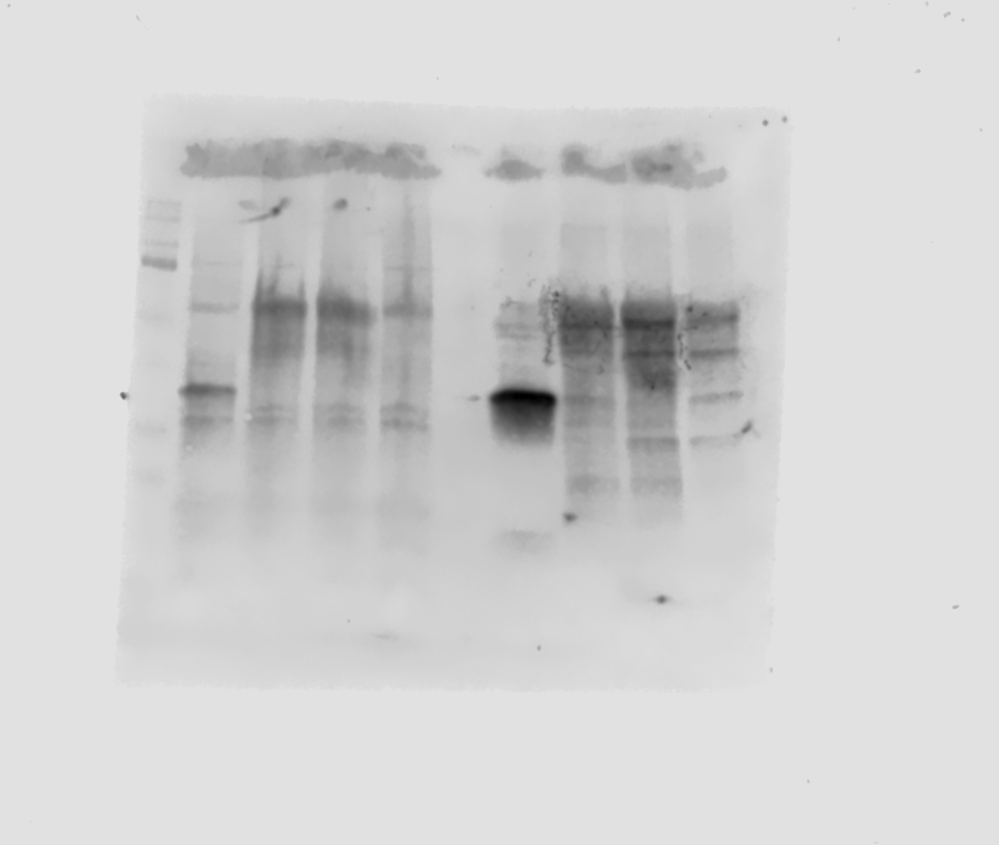

Supplement: Figure 3—source data 1. [file elife-82244-fig3-data1.zip › Source Data Figure 3/Source Data Figure 3I Flag.png]

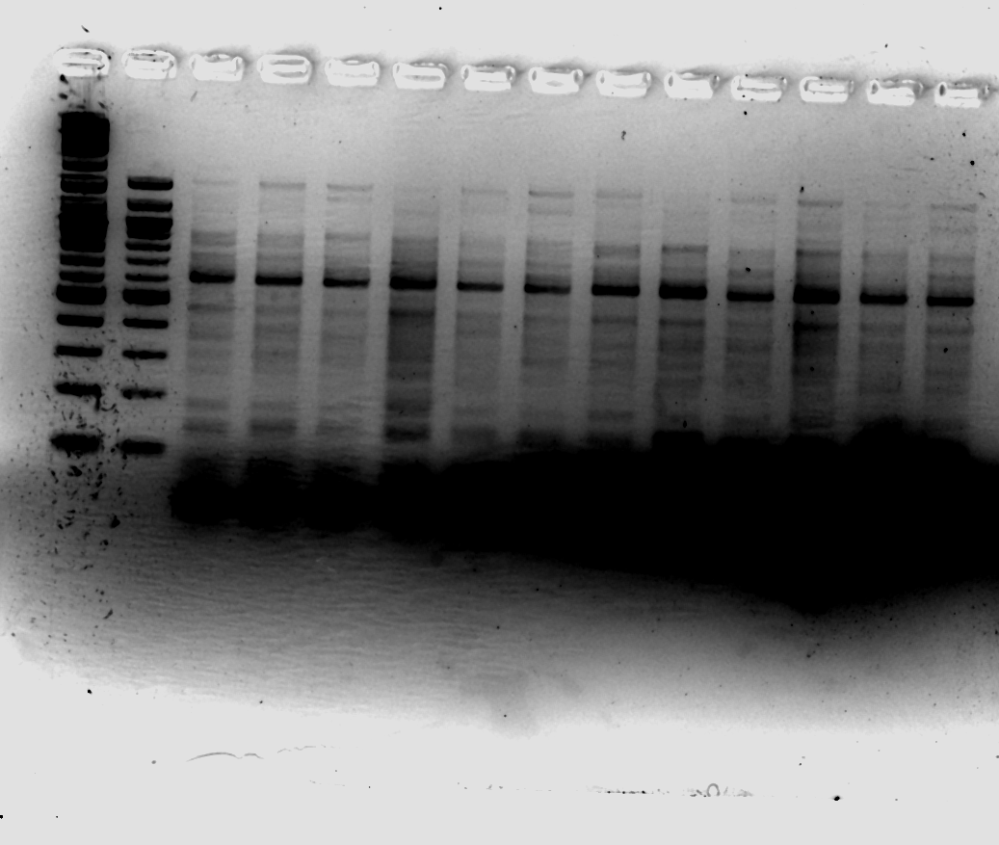

Supplement: Figure 3—figure supplement 1—source data 1. — Boxed bands indicate what is shown in the figure. Arrows indicate bands of interest. [file elife-82244-fig3-figsupp1-data1.zip › Source Data Figure 3 supplemental A.png]

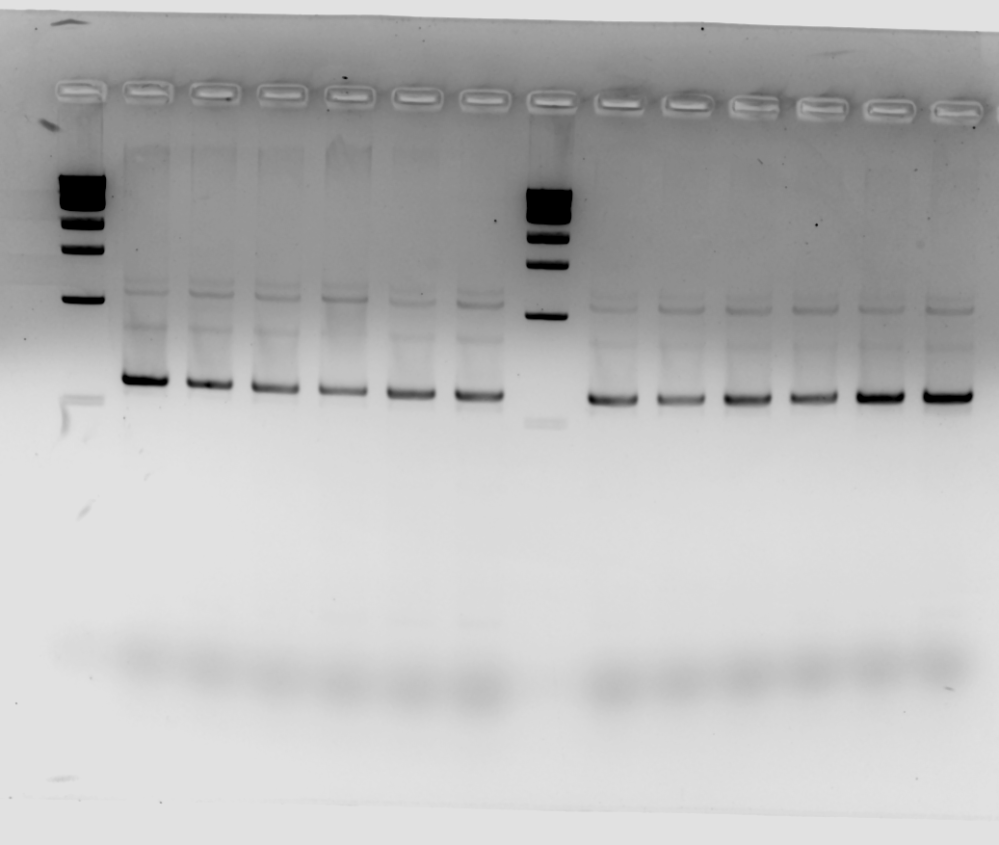

Supplement: Figure 3—figure supplement 1—source data 2. — Unmodified CLIP immunoblot of 3xFLAG-GFP and 3xFLAG-SRSF6 constructs expressed in RAW MΦ for 24 h. [file elife-82244-fig3-figsupp1-data2.zip › Source Data Figure 3 supplemental B Bax.png]

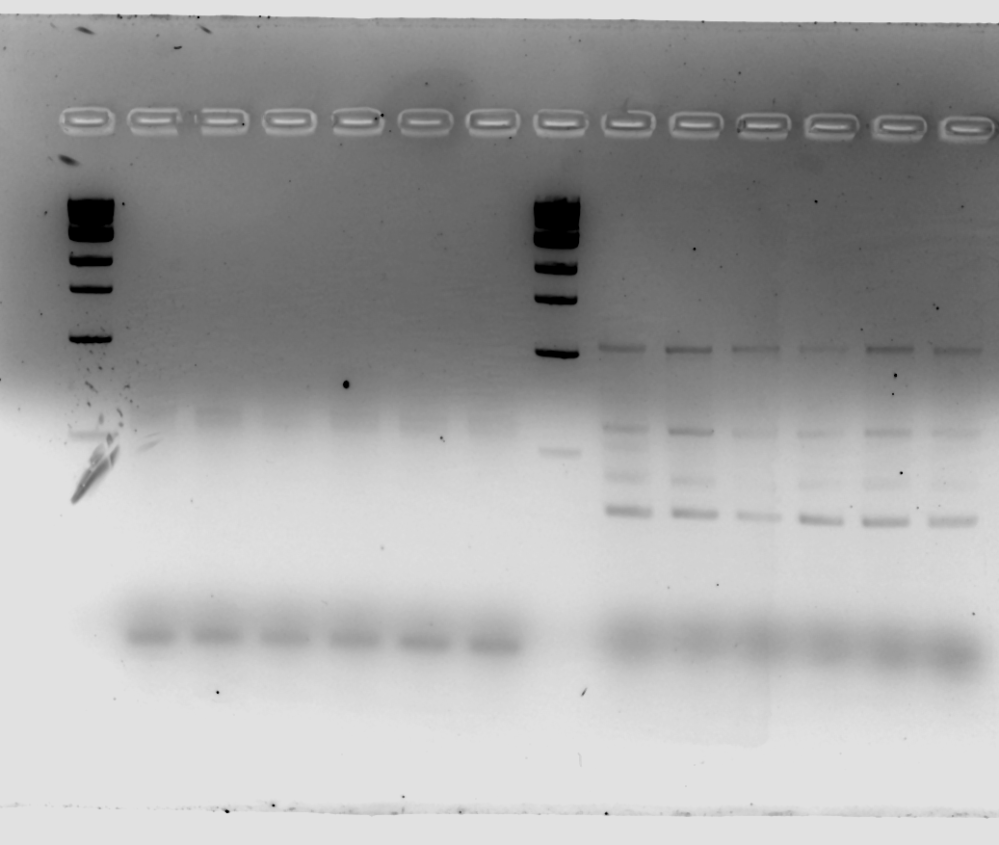

Supplement: Figure 3—figure supplement 1—source data 2. — Unmodified CLIP immunoblot of 3xFLAG-GFP and 3xFLAG-SRSF6 constructs expressed in RAW MΦ for 24 h. [file elife-82244-fig3-figsupp1-data2.zip › Source Data Figure 3 supplemental B Brd2.png]

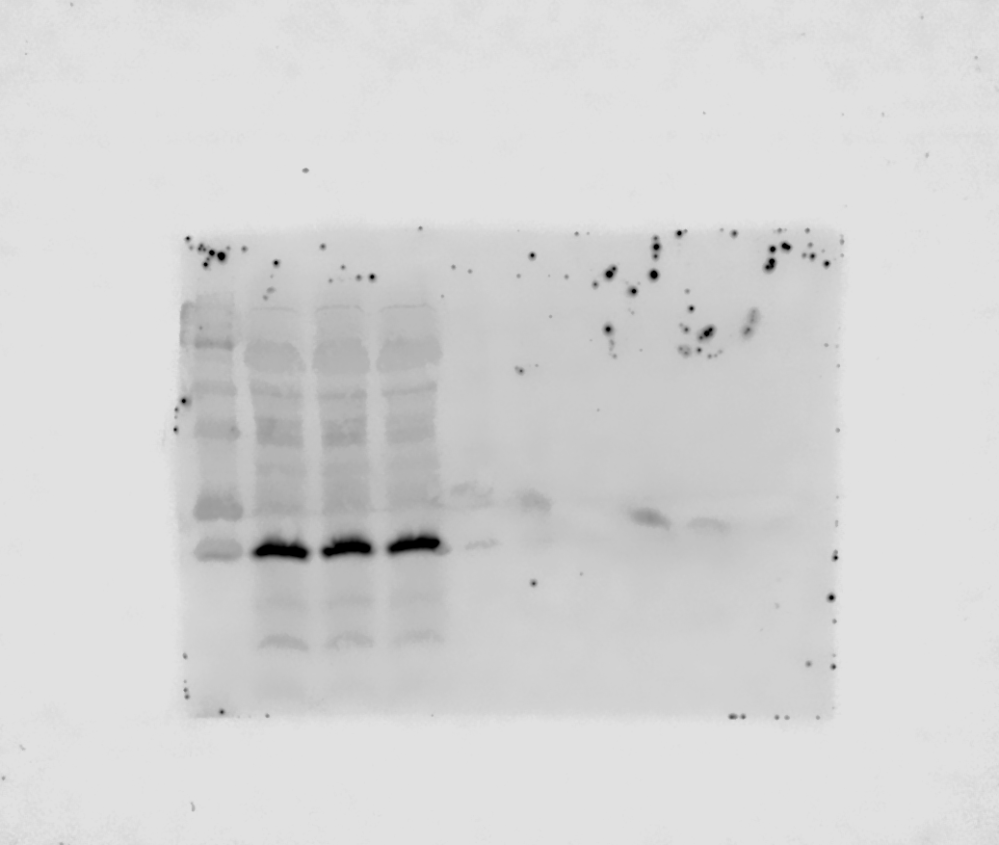

Supplement: Figure 3—figure supplement 1—source data 3. [file elife-82244-fig3-figsupp1-data3.zip › Source Data Figure 3 supplemental C Bax.png]

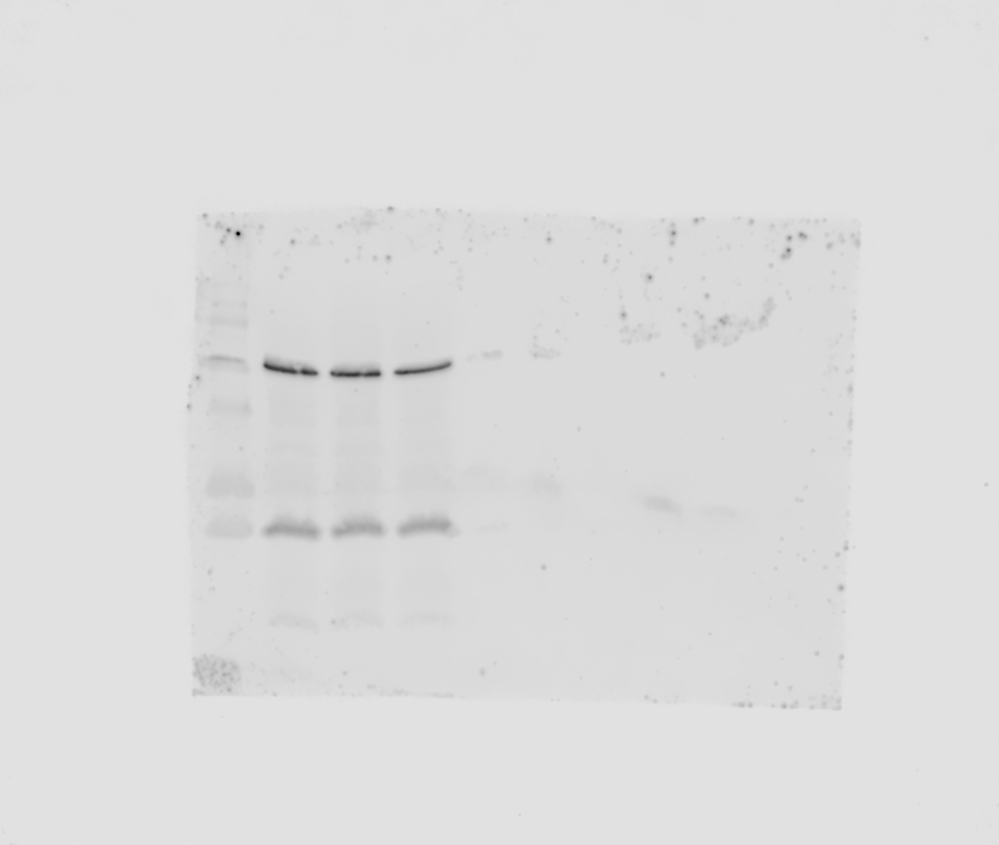

Supplement: Figure 3—figure supplement 1—source data 3. [file elife-82244-fig3-figsupp1-data3.zip › Source Data Figure 3 supplemental C Tubulin.png]

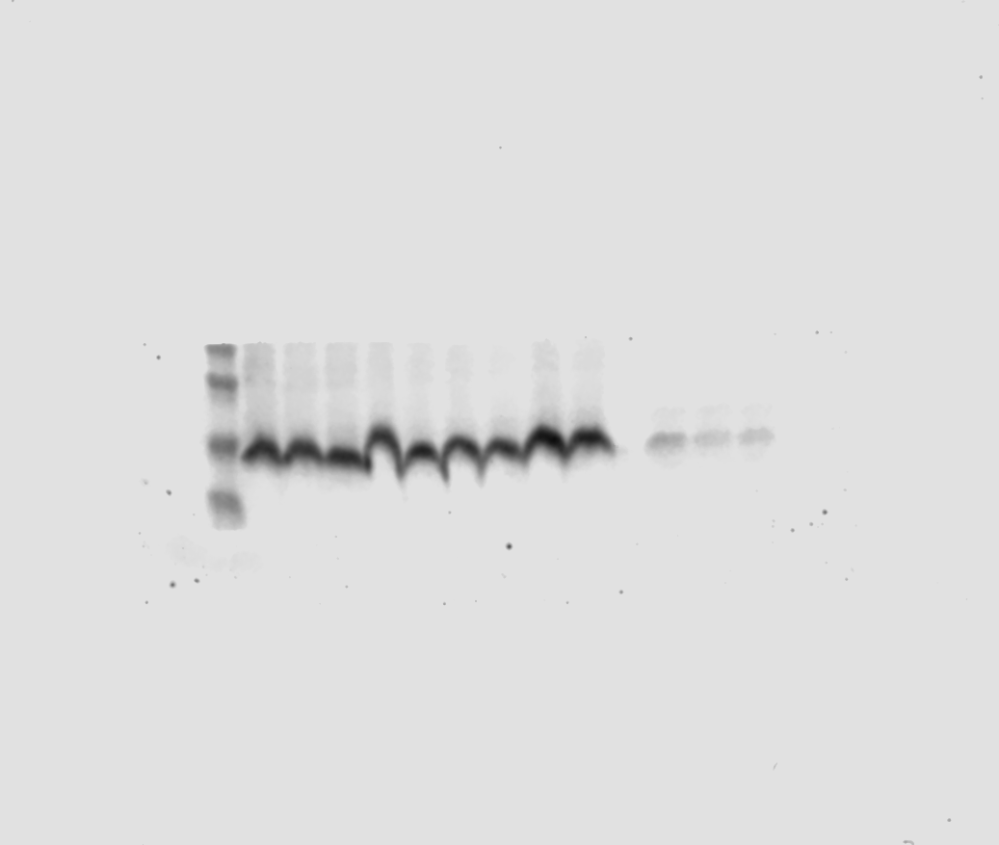

Supplement: Figure 4—source data 1. — SCR cells treated with staurosporine for 24 hr used as a positive control. Boxed bands indicate what is shown in the figure. Arrows indicate bands of interest. [file elife-82244-fig4-data1.zip › Source Data Figure 4/Source Data Figure 4I Mem cytochrome c.png]

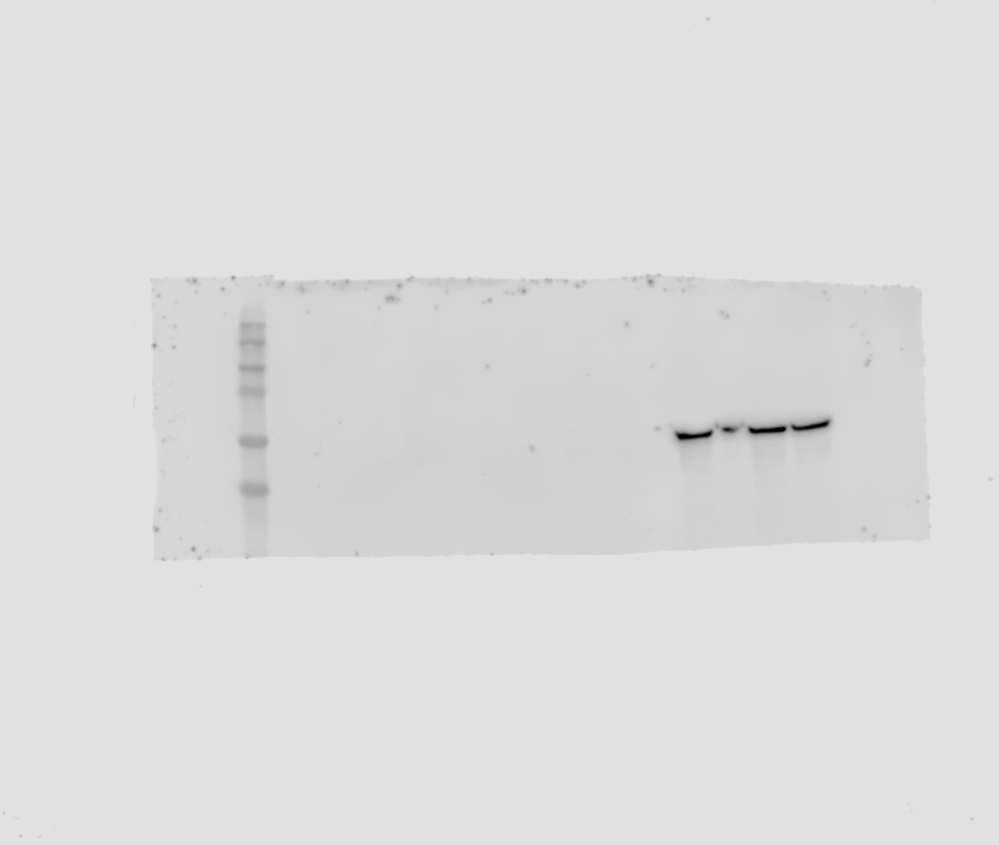

Supplement: Figure 4—source data 1. — SCR cells treated with staurosporine for 24 hr used as a positive control. Boxed bands indicate what is shown in the figure. Arrows indicate bands of interest. [file elife-82244-fig4-data1.zip › Source Data Figure 4/Source Data Figure 4I Cyto ATP5A1.png]

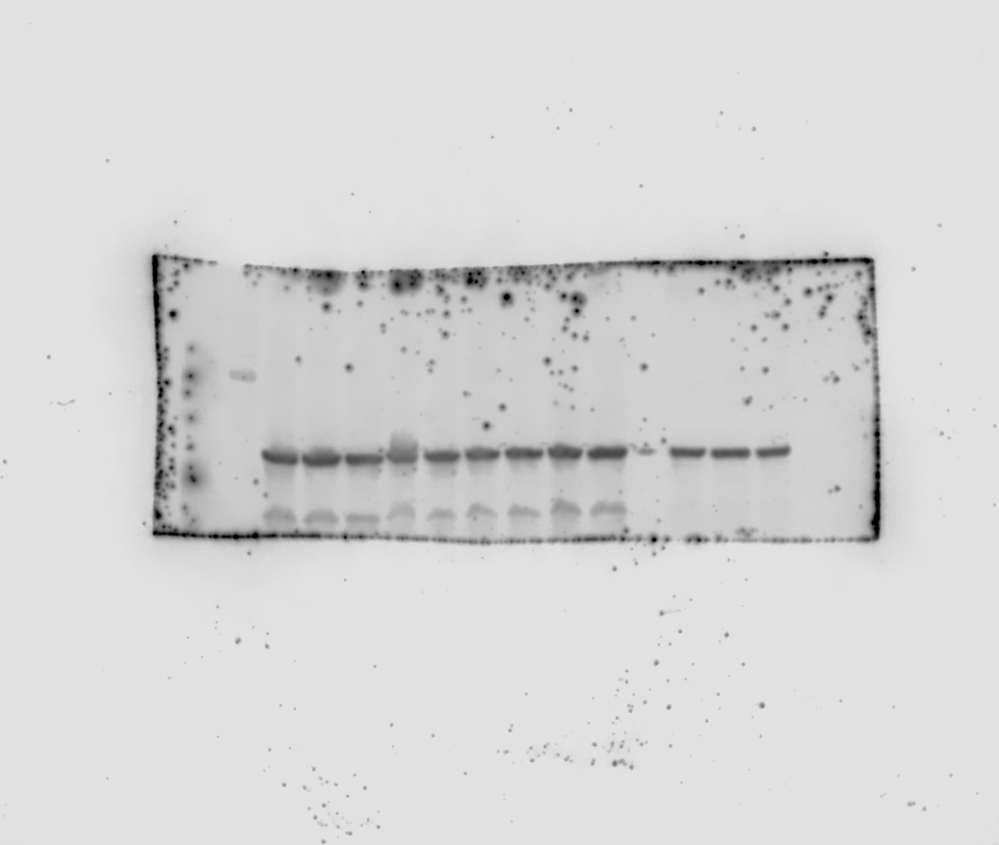

Supplement: Figure 4—source data 1. — SCR cells treated with staurosporine for 24 hr used as a positive control. Boxed bands indicate what is shown in the figure. Arrows indicate bands of interest. [file elife-82244-fig4-data1.zip › Source Data Figure 4/Source Data Figure 4I Mem Actin.png]

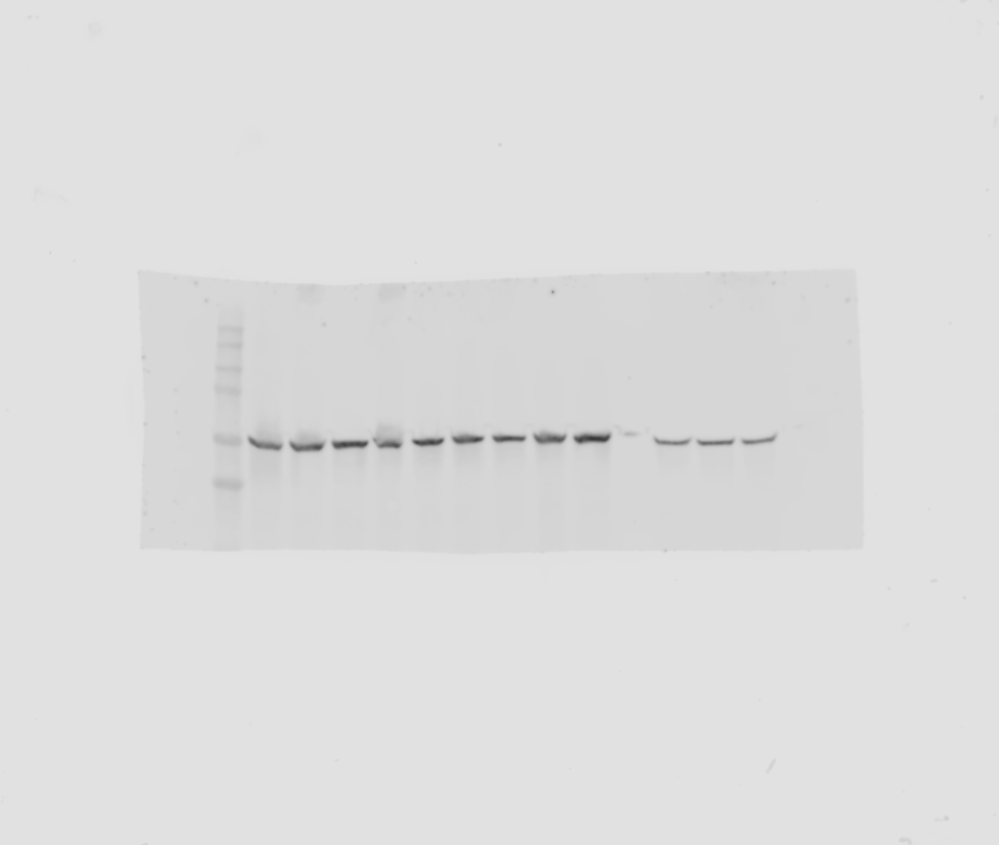

Supplement: Figure 4—source data 1. — SCR cells treated with staurosporine for 24 hr used as a positive control. Boxed bands indicate what is shown in the figure. Arrows indicate bands of interest. [file elife-82244-fig4-data1.zip › Source Data Figure 4/Source Data Figure 4I Mem ATP5A1.png]

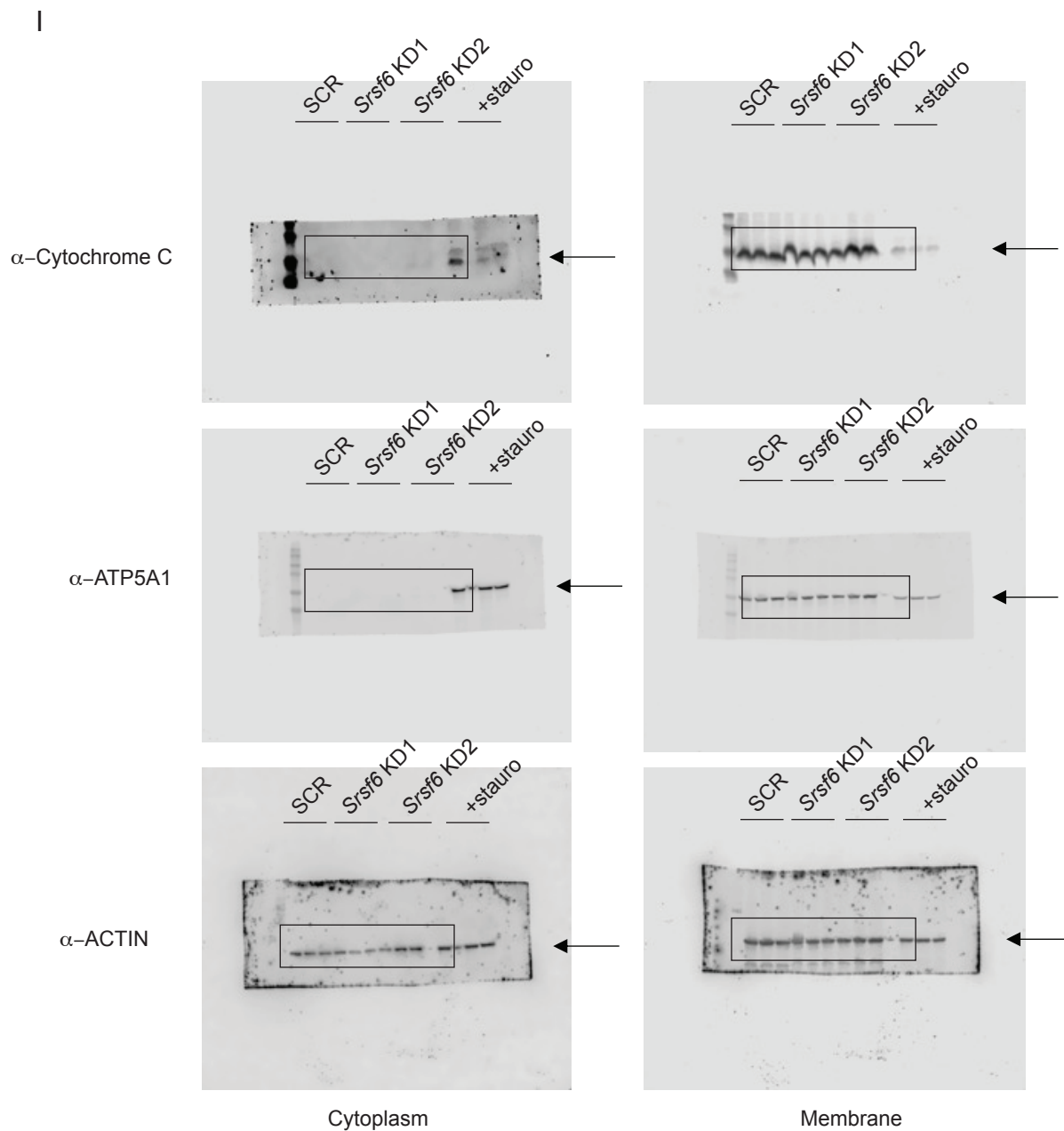

Figure 4. Source Data

Supplement: Figure 4—source data 1. — SCR cells treated with staurosporine for 24 hr used as a positive control. Boxed bands indicate what is shown in the figure. Arrows indicate bands of interest. [file elife-82244-fig4-data1.zip › Source Data Figure 4/eLIFE Figure 4, Source Data 1.pdf]

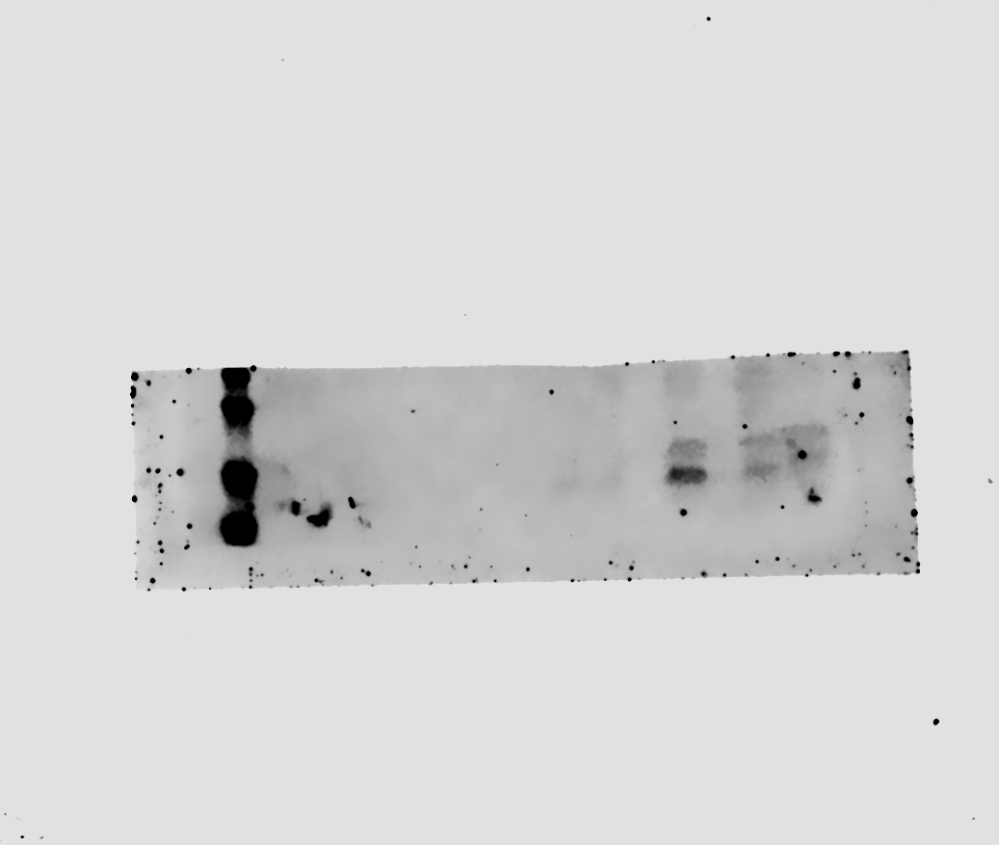

Supplement: Figure 4—source data 1. — SCR cells treated with staurosporine for 24 hr used as a positive control. Boxed bands indicate what is shown in the figure. Arrows indicate bands of interest. [file elife-82244-fig4-data1.zip › Source Data Figure 4/Source Data Figure 4I Cyto cytochrome c.png]

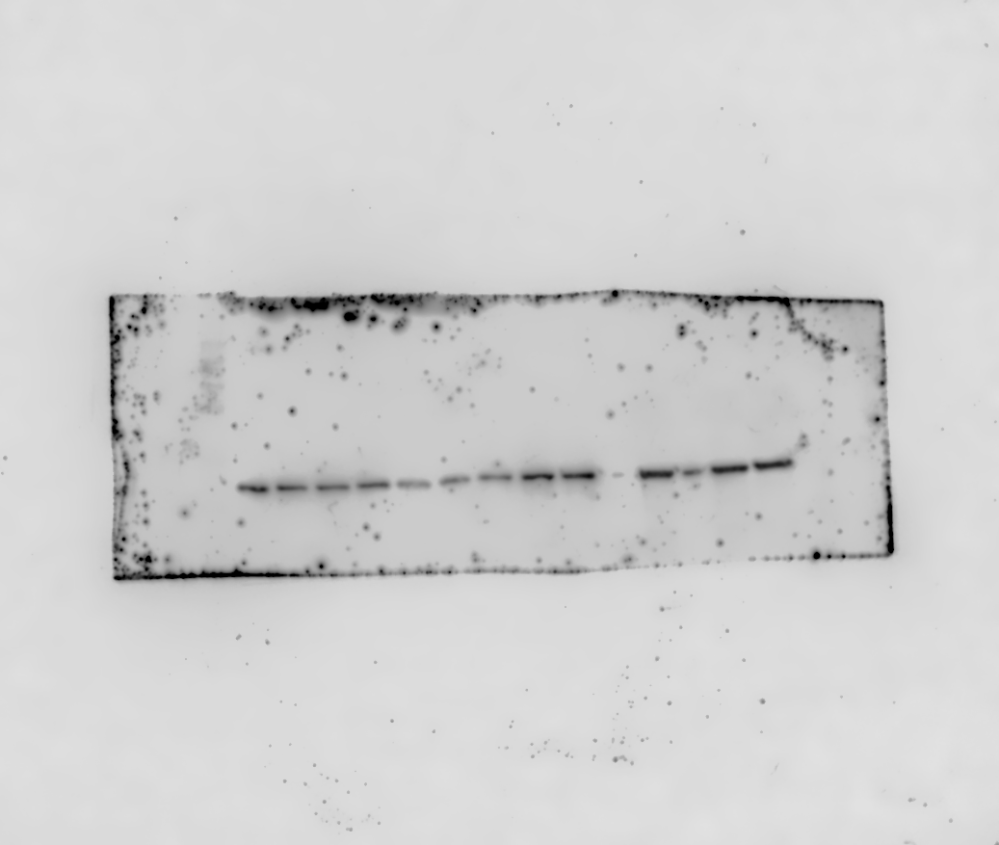

Supplement: Figure 4—source data 1. — SCR cells treated with staurosporine for 24 hr used as a positive control. Boxed bands indicate what is shown in the figure. Arrows indicate bands of interest. [file elife-82244-fig4-data1.zip › Source Data Figure 4/Source Data Figure 4I Cyto Actin.png]

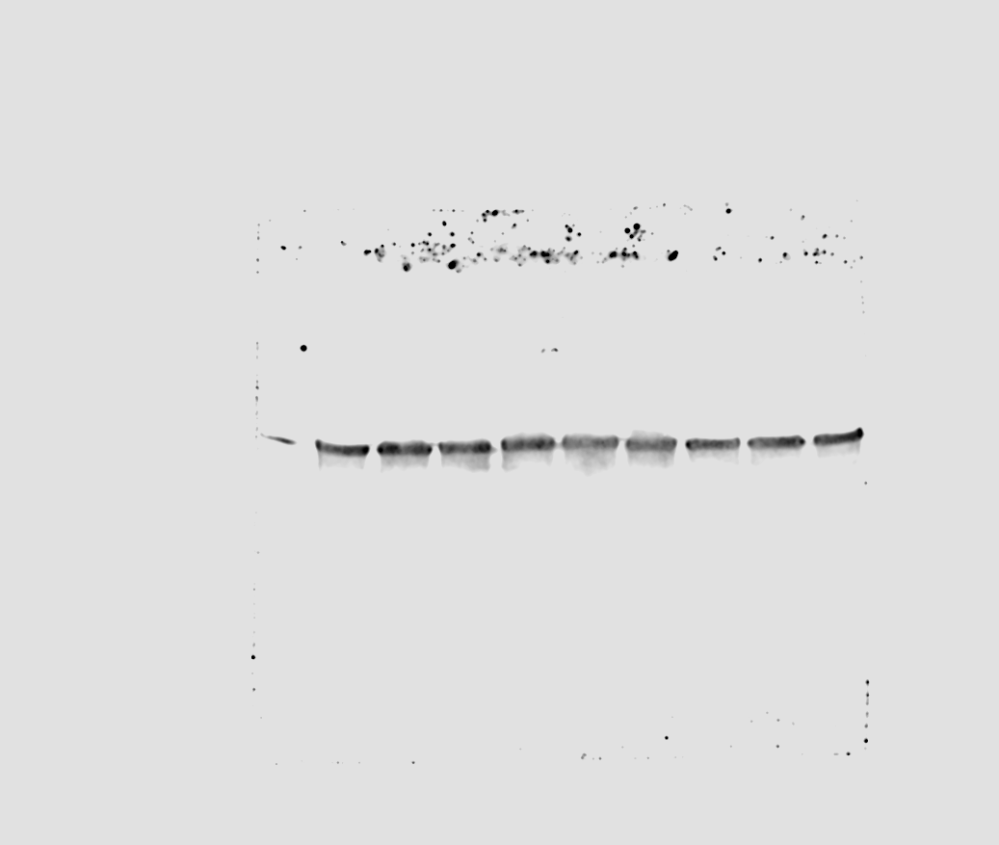

Supplement: Figure 5—source data 1. — Boxed bands indicate what is shown in the figure. Arrows indicate bands of interest. [file elife-82244-fig5-data1.zip › Source Data Figure 5/Source Data Figure 5A Bax Actin.png]

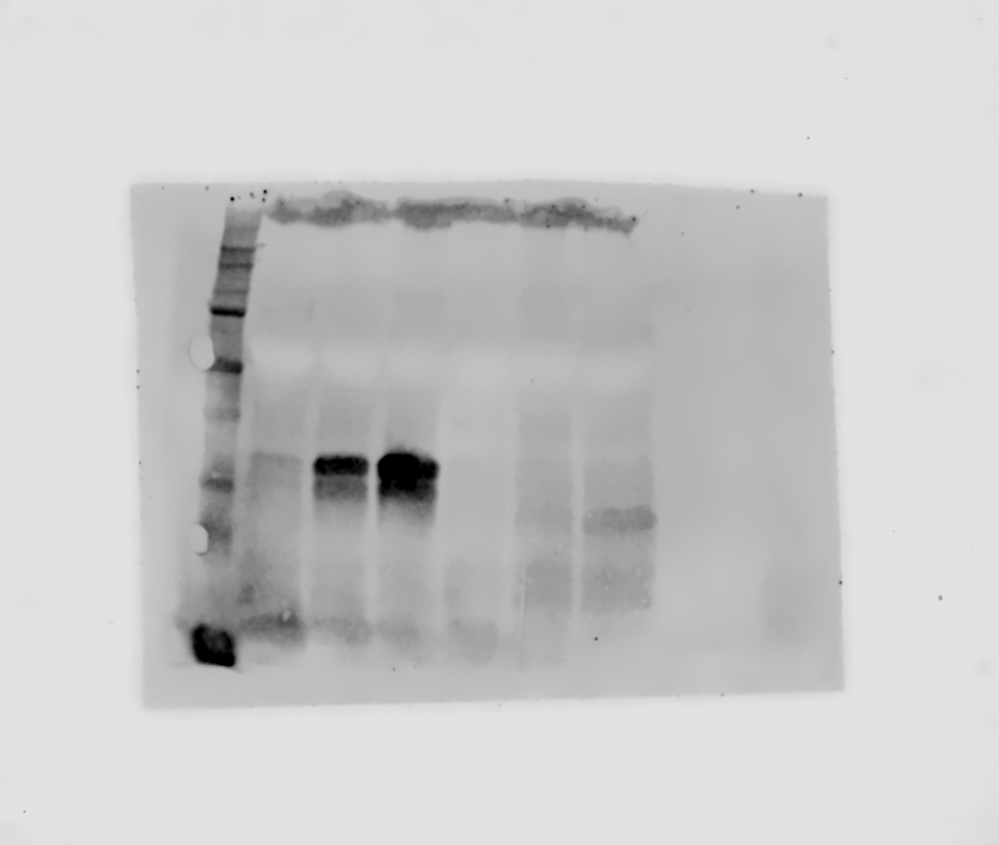

Supplement: Figure 5—source data 1. — Boxed bands indicate what is shown in the figure. Arrows indicate bands of interest. [file elife-82244-fig5-data1.zip › Source Data Figure 5/Source Data Figure 5A Baxkappa Strep.png]

A

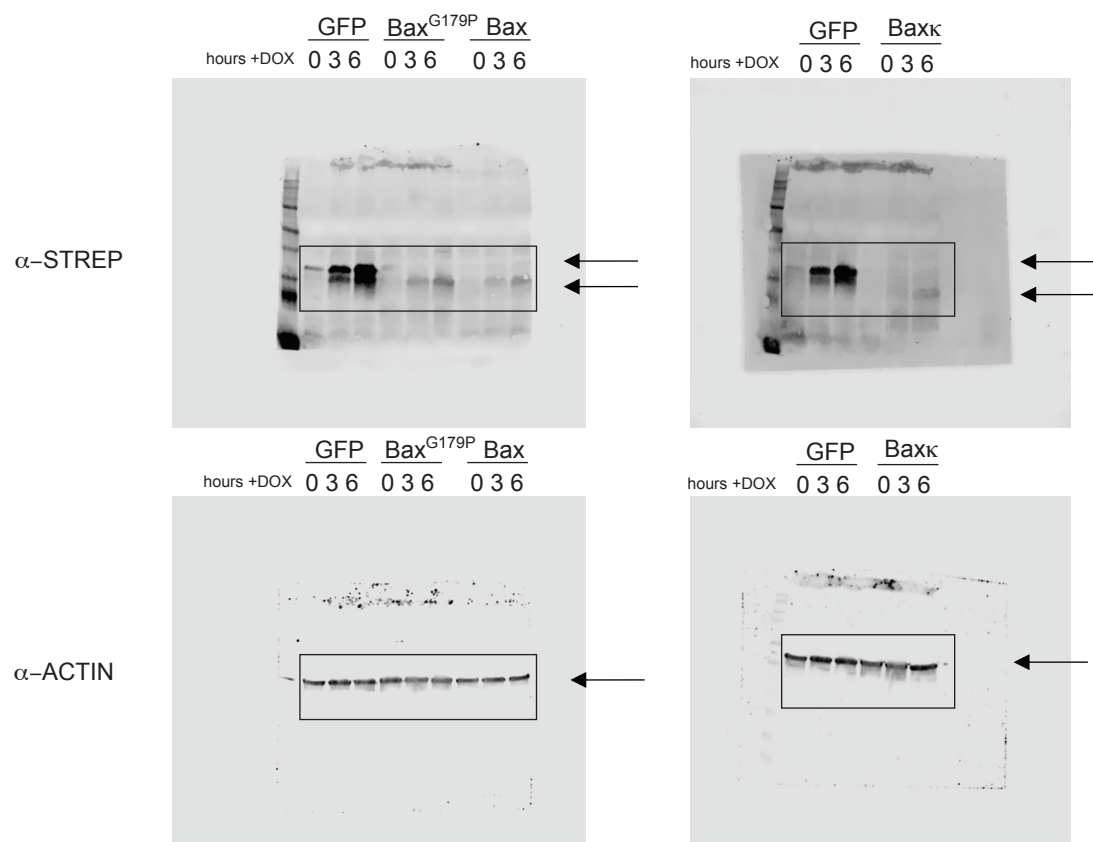

Figure 5. Source Data

Supplement: Figure 5—source data 1. — Boxed bands indicate what is shown in the figure. Arrows indicate bands of interest. [file elife-82244-fig5-data1.zip › Source Data Figure 5/eLIFE Figure 5, Source Data 1.pdf]

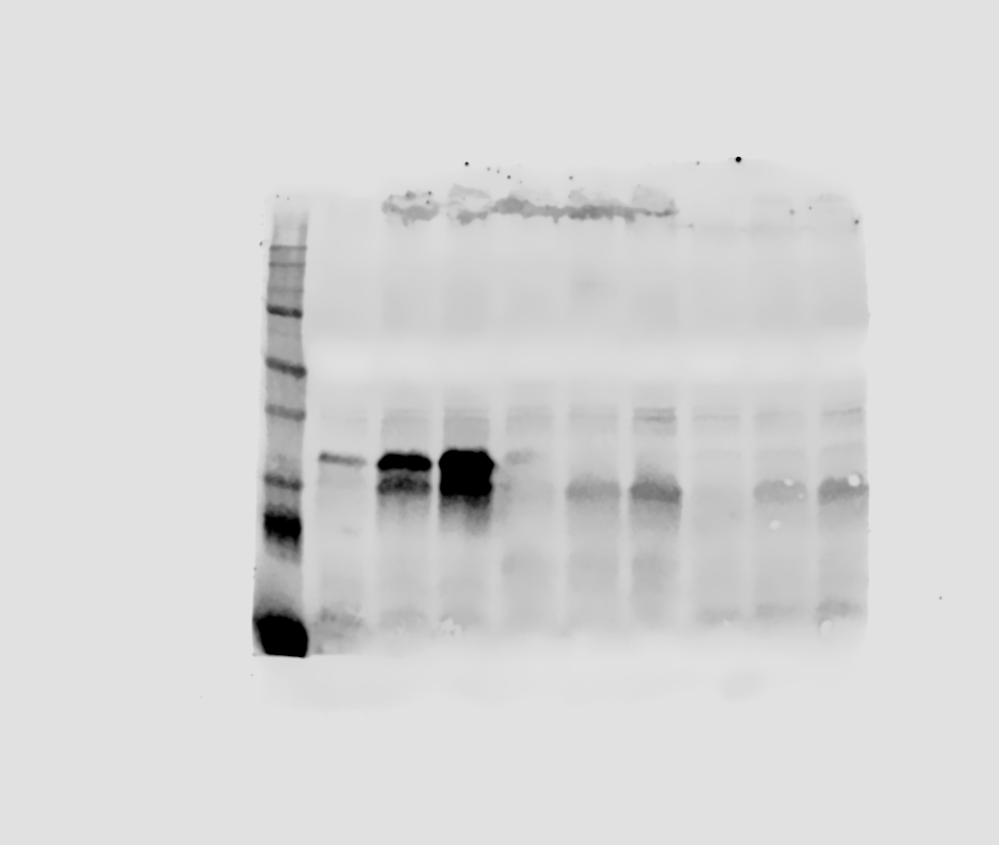

Supplement: Figure 5—source data 1. — Boxed bands indicate what is shown in the figure. Arrows indicate bands of interest. [file elife-82244-fig5-data1.zip › Source Data Figure 5/Source Data Figure 5A Bax Strep.png]

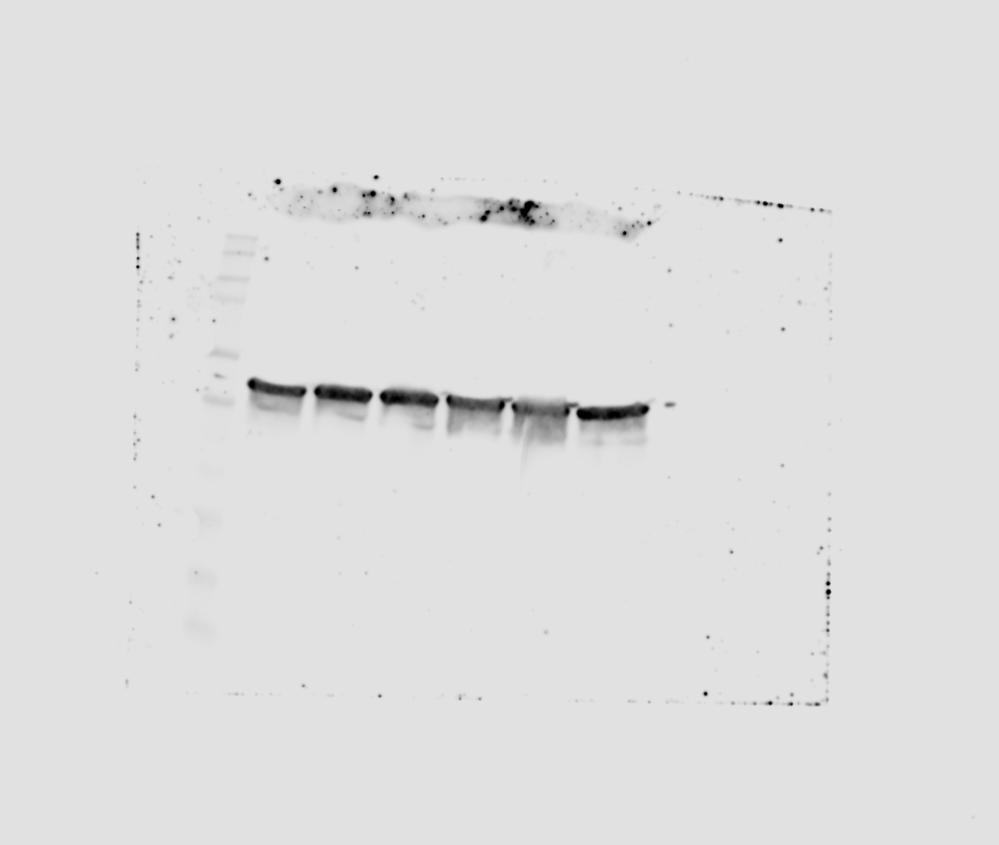

Supplement: Figure 5—source data 1. — Boxed bands indicate what is shown in the figure. Arrows indicate bands of interest. [file elife-82244-fig5-data1.zip › Source Data Figure 5/Source Data Figure 5A Baxkappa Actin.png]

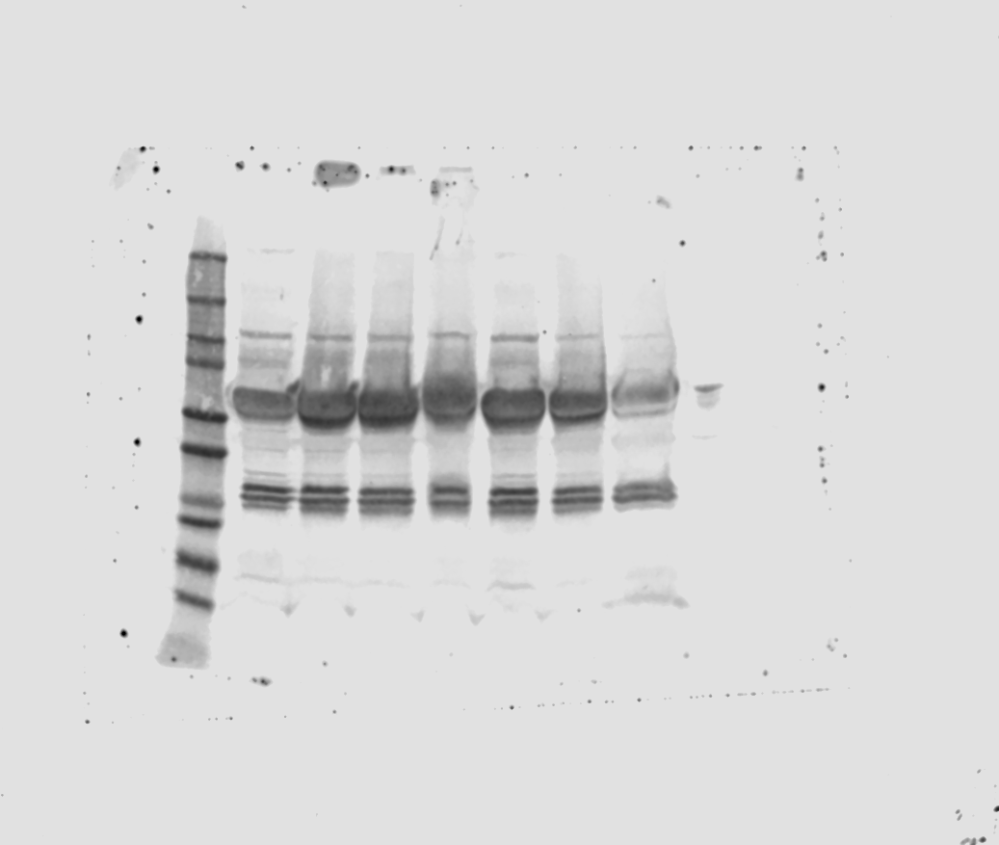

Supplement: Figure 6—source data 1. — Unmodified semi-quantitative RT-PCR gel of Bax and Brd2 (control) in FLAG-tagged SRSF6, SRSF6S303A, and SRSF6S303D inducible RAW MΦ expressed for 24 h after DOX induction. Boxed bands indicate what is shown in the figure. Arrows indicate bands of interest. [file elife-82244-fig6-data1.zip › Source Data Figure 6/Source Data Figure 6B Flag.png]

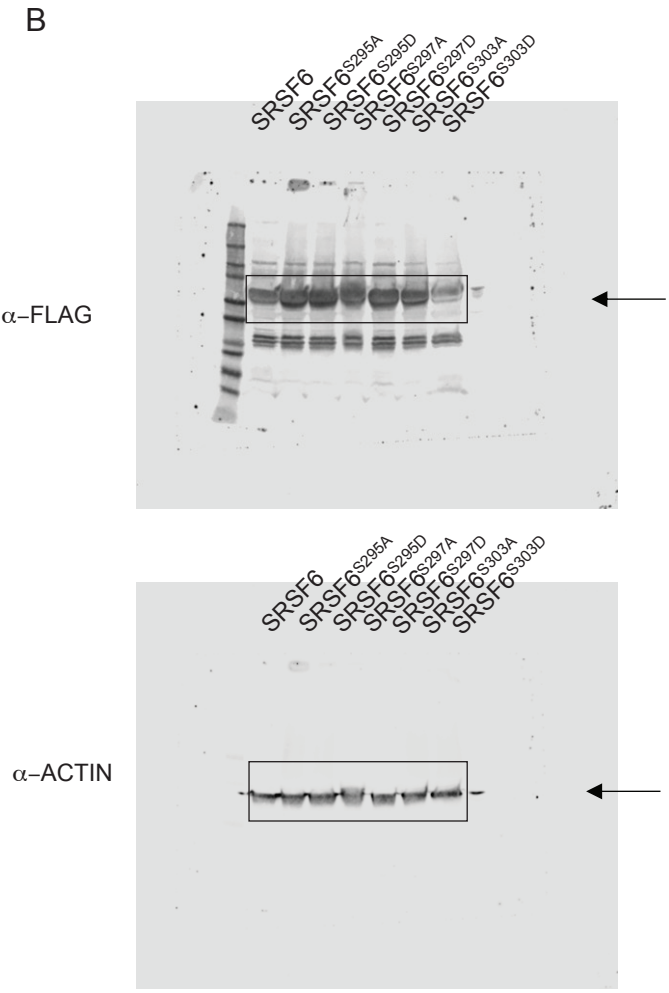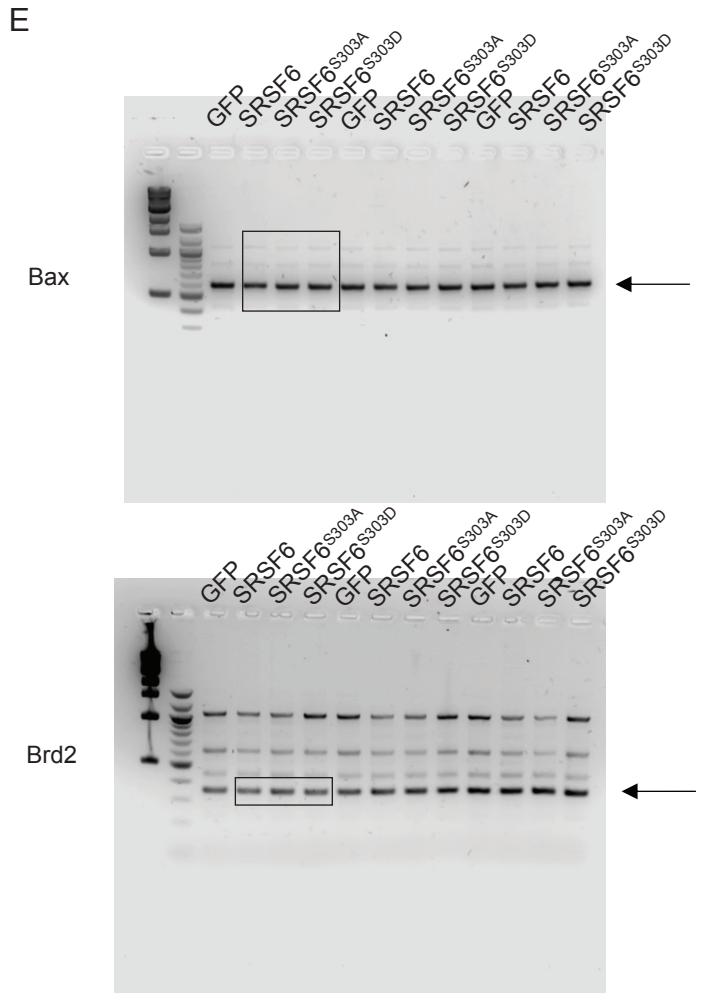

Figure 6. Source Data

Supplement: Figure 6—source data 1. — Unmodified semi-quantitative RT-PCR gel of Bax and Brd2 (control) in FLAG-tagged SRSF6, SRSF6S303A, and SRSF6S303D inducible RAW MΦ expressed for 24 h after DOX induction. Boxed bands indicate what is shown in the figure. Arrows indicate bands of interest. [file elife-82244-fig6-data1.zip › Source Data Figure 6/eLIFE Figure6 Source Data_New.pdf]

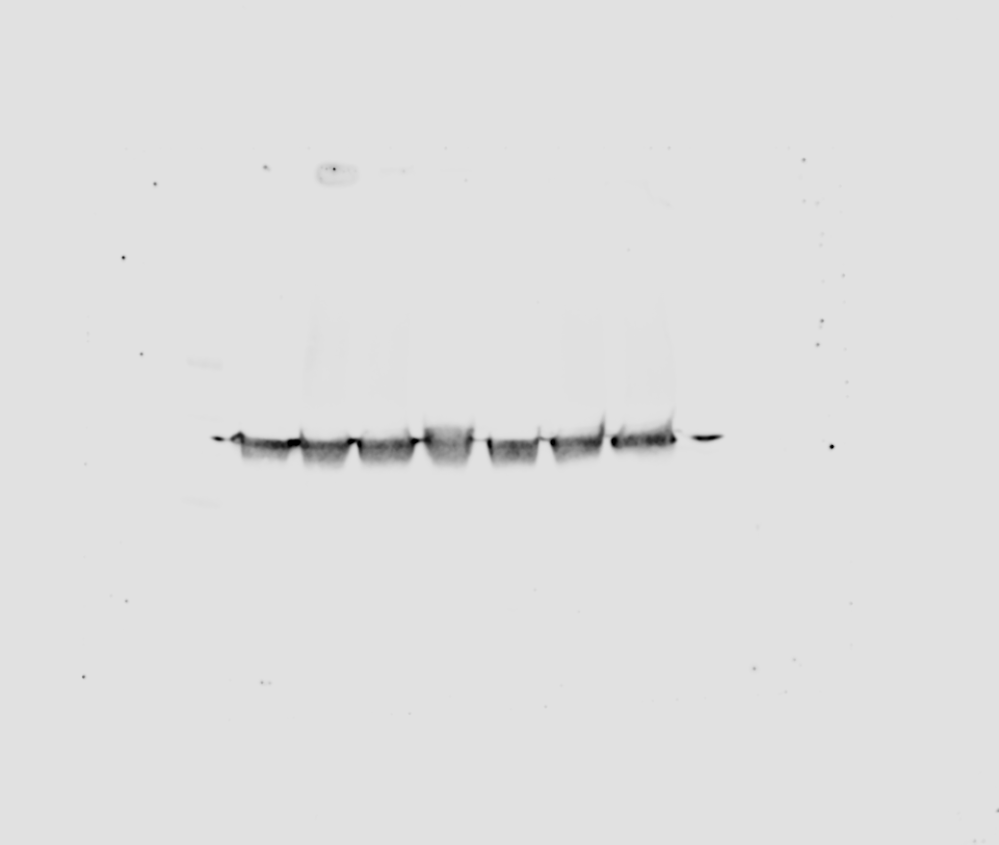

Supplement: Figure 6—source data 1. — Unmodified semi-quantitative RT-PCR gel of Bax and Brd2 (control) in FLAG-tagged SRSF6, SRSF6S303A, and SRSF6S303D inducible RAW MΦ expressed for 24 h after DOX induction. Boxed bands indicate what is shown in the figure. Arrows indicate bands of interest. [file elife-82244-fig6-data1.zip › Source Data Figure 6/Source Data Figure 6B Actin .png]

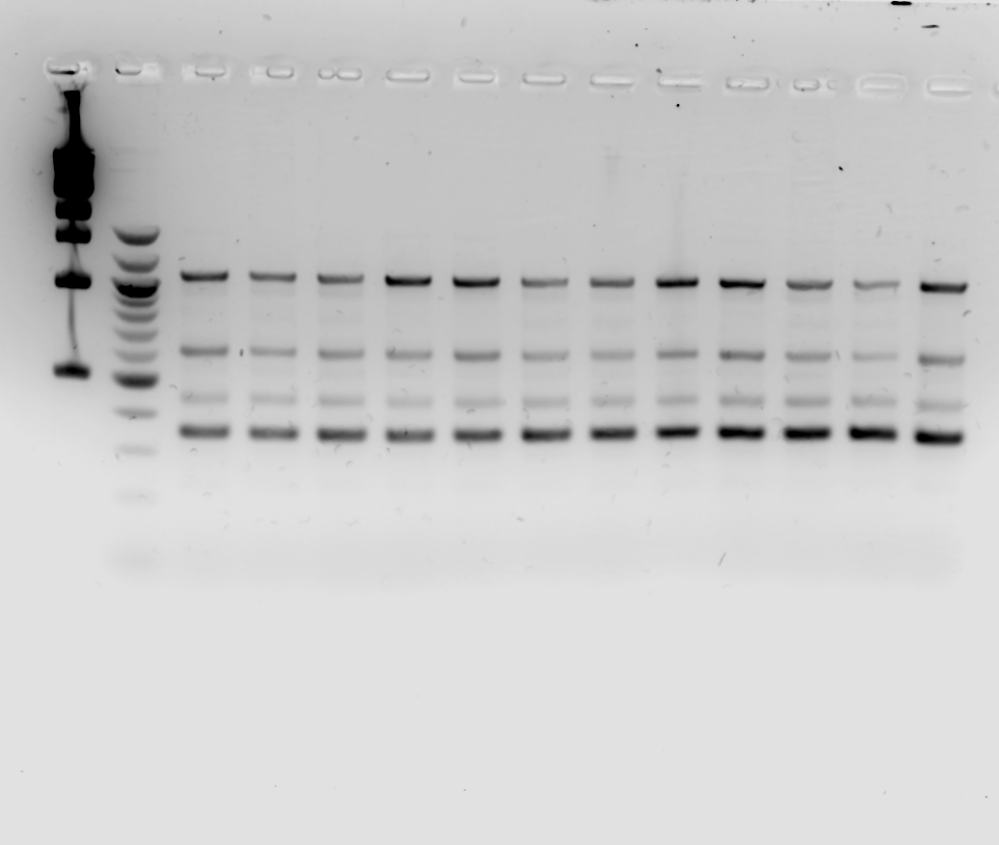

Supplement: Figure 6—source data 1. — Unmodified semi-quantitative RT-PCR gel of Bax and Brd2 (control) in FLAG-tagged SRSF6, SRSF6S303A, and SRSF6S303D inducible RAW MΦ expressed for 24 h after DOX induction. Boxed bands indicate what is shown in the figure. Arrows indicate bands of interest. [file elife-82244-fig6-data1.zip › Source Data Figure 6/Source Data Figure 6E Brd2.png]

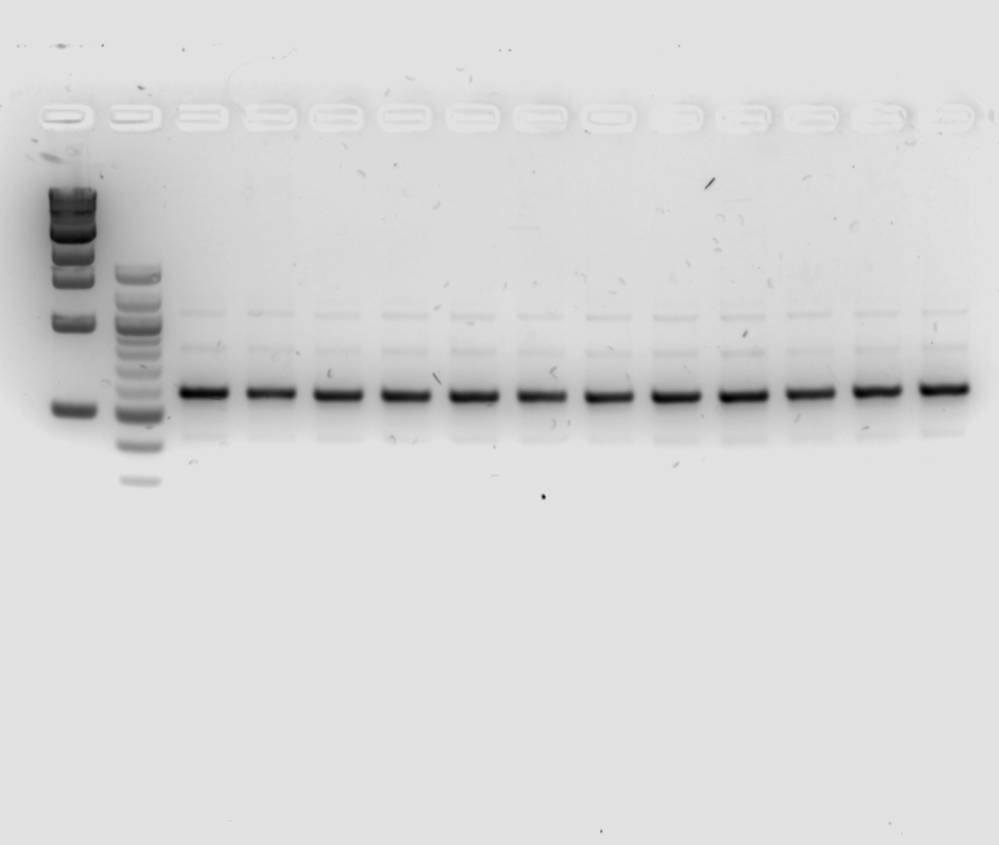

Supplement: Figure 6—source data 1. — Unmodified semi-quantitative RT-PCR gel of Bax and Brd2 (control) in FLAG-tagged SRSF6, SRSF6S303A, and SRSF6S303D inducible RAW MΦ expressed for 24 h after DOX induction. Boxed bands indicate what is shown in the figure. Arrows indicate bands of interest. [file elife-82244-fig6-data1.zip › Source Data Figure 6/Source Data Figure 6E Bax.png]
